# Maternal and perinatal death surveillance and response in Ethiopia: Achievements, challenges and prospects

**DOI:** 10.1371/journal.pone.0223540

**Published:** 2019-10-11

**Authors:** Brhane Ayele, Hailay Gebretnsae, Tsegay Hadgu, Degnesh Negash, Fana G/silassie, Tesfu Alemu, Esayas Haregot, Tewolde Wubayehu, Hagos Godefay

**Affiliations:** 1 Tigray Health Research Institute, Mekelle, Tigray, Ethiopia; 2 UNFPA-Tigray, Ethiopia; 3 Tigray Regional Health Bureau, Mekelle, Tigray, Ethiopia; United Nations Population Fund, BANGLADESH

## Abstract

**Background:**

Maternal and Perinatal Death Surveillance and Response (MPDSR) was a pilot program introduced in Tigray, Ethiopia to monitor maternal and perinatal death. However; its implementation and operation is not evaluated yet. Therefore, this study aimed to assess the implementation and operational status and determinants of MPDSR using a programmatic data and stakeholders involved in the program.

**Methods:**

Institutional based cross-sectional study was applied in public health facilities (75 health posts, 50 health centers and 16 hospitals) using both qualitative and quantitative methods. Data were entered in to Epi-info and then transferred to SPSS version 21 for analysis. All variables with a p-value of ≤ 0.25 in the bivariate analysis were included in to multivariable logistic regression model to identify the independent predictors. For the qualitative part, manual thematic content analysis was done following data familiarization (reading and re-reading of the transcripts).

**Results:**

In this study, only 34 (45.3%) of health posts were practicing early identification and notification of maternal/perinatal death. Furthermore, only 36 (54.5%) and 35(53%) of health facilities were practiced good quality of death review and took proper action respectively following maternal/perinatal deaths. Availability of three to four number of Health Extension Workers (HEWs) (Adjusted Odds Ratio (AOR) = 6.09, 95%CI (Confidence Interval): 1.51–24.49), availability of timely Public Health Emergency Management (PHEM) reports (AOR = 4.39, 95%CI: 1.08–17.80) and participation of steering committee’s in death response (AOR = 9.19, 95%CI: 1.31–64.34) were the predictors of early identification and notification of maternal and perinatal death among health posts. Availability of trained nurse (AOR = 3.75, 95%CI: 1.08–12.99) and health facility’s head work experience (AOR = 3.70, 95%CI: 1.04–13.22) were also the predictors of quality of death review among health facilities. Furthermore; availability of at least one cluster review meeting (AOR = 4.87, 95%CI: 1.30–18.26) and uninterrupted pregnant mothers registration (AOR = 6.85, 95%CI: 1.22–38.54) were associated with proper response implementation to maternal and perinatal death. Qualitative findings highlighted that perinatal death report was so neglected. Community participation and intersectoral collaboration were among the facilitators for MPDSR implementation while limited human work force capacity and lack of maternity waiting homes were identified as some of the challenges for proper response implementation.

**Conclusion:**

This study showed that the magnitude of: early death identification and notification, review and response implementation were low. Strengthening active surveillance with active community participation alongside with strengthening capacity building and recruitment of additional HEWs with special focus to improve the quality of health service could enhance the implementation of MPDSR in the region.

## Introduction

The global maternal mortality ratio has been reduced significantly (by 43.3%) over the last fifteen years from 385 to 216 deaths per 100,000 live births. However, it still continues to be a public health challenge in low resource countries where 10.7 million women died since 1990 [1, 2].

Sub-Saharan Africa (SSA) is the hardest hit region and with unsatisfactory reduction over the previous years [3]. Compared to other regions, maternal mortality remains to be one of the highest (450 deaths per 100, 000) in SSA [4]. Maternal mortality in SSA varies between countries. For instance, maternal mortality in Nigeria was reported to be 448 per 100,000 live births [5] and institutional maternal death of 281 deaths per 100, 000 births [6] in Mozambique. Furthermore, another study conducted in 10 various SSA countries also reported 351 maternal deaths per 100, 000 live births [7]. Maternal mortality in Malawi was also reported to be 484 deaths per 100, 000 live births [8]. There is also big variation in, maternal mortality with in countries which ranged between 250 to 700 death per 100, 000 live births [9].

The major causes of maternal mortality worldwide are hemorrhage, hypertensive disorder and sepsis [10]. Similarly, hemorrhage, hypertensive disorder, abortion and obstructed labour are the major cause of maternal mortality in SSA [11–13]. Various factors such as socio-demographic factors and other health system related factors are distant causes that contribute to maternal mortality [14]. In addition, factors such as lack of skilled birth attendants, countries economic growth, health expenditure per capital are fundamental determinants of maternal mortality in a country [8, 15]. For instance, a study by Colbourn, T. et al, argued that increasing government spending on the health system, improved female literacy and education which play a vital role in reducing maternal mortality [8].

Perinatal mortality in SSA is also one of the highest where 70 and 56 deaths per 1000 births for home and health facility deliveries were reported respectively [16]. There is slight difference between countries in perinatal mortality. For instance, perinatal mortality in Burkina Faso is reported to be 79 deaths per 1000 live births [17].

Early neonatal mortality in SSA is worrisome. For instance a study from Burkina Faso reported 27 per 1000 live births [17] while another study from Nigeria found 32 deaths per 1000 live births [18] and 28 death per 1000 births [19]. Majority of under-five mortality occur during infancy [20, 21]. A Study reported that low birth weight, delivering outside of health facility, lack of attendant at health facilities, being attended by traditional birth attendant were the causes for early neonatal death [21].

Most of maternal and perinatal related deaths can be averted, even in resource-poor countries but, it requires the right kind of information for decision making. Empirical evidence is very vital in the fight against maternal, perinatal and neonatal mortality where specific context based interventions are fundamental to significantly reduced maternal, perinatal and neonatal mortality. However, the true burden of maternal deaths, stillbirths and neonatal deaths has been unknown and unavailable, and estimation of maternal, perinatal and neonatal mortality needs a big national survey which is unaffordable for countries. Although women and their babies share the same period of highest risk, often with the same health workers present, less information has been captured for stillbirths and neonatal deaths than for maternal deaths [22]. Improving measurement is the first step to understand where action is needed. It is possible to establish a system to assess the burden of maternal mortality, stillbirths and neonatal deaths [23]. Some of the strategies to measure maternal, perinatal and neonatal mortality are; death notification for maternal and neonatal deaths and stillbirths in the community and facility, death review for maternal and neonatal deaths to identify the causes, gaps, and challenges that need to overcome [24].

Knowing the statistics on levels of mortality is not enough; information that helps to identify what can be done to prevent such unnecessary deaths is also highly important [23]. Precise estimation of maternal mortality is helpful to design important and cost effective interventions to reduce maternal, perinatal and neonatal mortality by improving quality of service [22].

Maternal and neonatal mortality has reduced significantly to 412 deaths per 100, 000 live births in Ethiopia despite regional and intra-regional variations [25]. Neonatal Mortality Ratio (NMR) and Perinatal Mortality Rate (PMR) were 29 and 33 per 1000 births respectively [25]. The current MMR and NMR status are still short of the country’s target of reducing maternal and neonatal mortality to 199/100,000 live births and 10/1000 live birth respectively by the year 2020 [26].

Various methods to measure maternal mortality are used in Ethiopia with different level of precision of the estimates. For instance, maternal mortality using verbal autopsy in Tigray region ranged from 37 to 482 death per 100, 000 live births demonstrating a big intra-regional difference [27]. Other methods used to estimate maternal mortality in Ethiopia is Maternal Death Surveillance and Response (MDSR). In Ethiopia, MDSR (Maternal Death Surveillance and Response) system was introduced in 2013 as a national policy to identify maternal mortality and foster local actions in the fight against maternal mortality [26, 28]. Since August 2017, the MDSR system was changed to Maternal and Perinatal Death Surveillance and Response (MPDSR) by adding the extended definition of perinatal death and in Tigray region, this programme was introduced in 2015 two years ahead of the national implementation [29]. MPDSR is a system established that enforce all health facilities and communities to report all maternal, perinatal and neonatal death to the next higher level of the health system. This enables to examine and identify the causes of maternal, perinatal, neonatal death and create awareness in the communities and health facilities to prevent and control preventable deaths.

Despite the importance of MPDSR in generating up to date evidence for decision making, evidence on the operation, implementation and inter facility differences is lacking. Some evidence are collected at regional level but it lacked comprehensiveness. Therefore, this study was aimed to assess the implementation status of MPDSR and its associated factors as well as explore the barriers and facilitators of MDSR implementation and operation in Tigray region, Northern Ethiopia.

## Methodology

### Study setting and period

Tigray region is one of the nine regional states of Ethiopia located in the North and the region is administratively structured into seven zones. Tigray region has 52 districts (34 rural and 18 urban) and 799 Kebeles (722 rural and 77 urban). Kebele is the lowest administrative unit in Ethiopia with an average population of 5000. In Tigray region, there are various health facilities that provide curative and promotive health services and average primary health care coverage is 91.7% [30] (**Table 1**). The study was conducted from December 1/2017 to January 30/2018.

**Table 1:** Type and number of health facilities found in Tigray region Northern Ethiopia 2019.

Type of health facility	Number	Remark
Specialized hospital	2	
General hospitals	15	
Primary hospitals	22	
Primary health care centers	216	
Health posts	712	
Private health facilities	500	Hospital, clinics, Medicals centers

### Study design

This study employed both quantitative and qualitative approaches. The quantitative approach was a community and health facility based (health post to hospital level) cross-sectional study design which was included the period of implementation from July/2016 to June/2017(one year performance). A qualitative study method using in-depth interviews and focus group discussions were also used to further explore barriers and facilitators of MDSR implementation and operation.

### Sampling procedures and participant recruitment

For the quantitative part of the study, using the annual regional maternal death list report, 50% of districts with at least one maternal death in the previous one year (for economic reasons) were randomly selected by stratifying them into three categories: districts with 4 and above maternal death as “high maternal death”, districts with 2–3 maternal death as, “medium maternal death” and districts with 1 maternal death as, “low maternal death”. Following this, all health facilities in the selected districts with maternal death (either community or health facility based death) were included. Then all health posts with maternal death and 50% of health posts with at least one reported community based perinatal death were included (**S1 Fig**).

For the qualitative part of the study, one focused group discussion (FGD) for each category (district with high, medium and low maternal death) (with 8–10 members each) was included with a total number of three FGDs. FGD participants were purposively sampled women development group leaders (WDGL) where they are supposed to be best performers of the various health packages. They were selected from the same village of the deceased mother for the purpose of receiving accurate information.

Additionally, a total of eleven in-depth interviews were conducted with health managers working in health facilities, district health offices and regional health bureau. Sample size for the FGD and in-depth interview participants were determined by data saturation. For the quantitative part of the study, health facility directors, supervisors of HEWs, HEWs, surveillance focal persons and heads of MCH department in the selected districts were interviewed to assess the implementation status of MPDSR.

### Data collection tool and technique

Two separated semi-structured questionnaires (i.e. for community (health post) and health facility level) were developed, in English language, based on the literatures mainly on MPDSR national and regional guidelines’ performance measure indicators. Finally, the questionnaires were given to experts who were working in Maternal, Neonatal and Child Health (MNCH) program for feedbacks and the data collection tool was revised accordingly.

The questionnaire for health post (**S1 File**) composed of three components:1) general information such as number of HEWs in the Kebele, Keble’s household size, HEW’s work experience, HEW’s educational level, HEW’s training status on MPDSR and availability of infrastructures in the Kebele–like road and networks; 2) surveillance and surveillance related characteristics such as availability of notification format, availability of death list (maternal and perinatal) format, uninterrupted pregnancy surveillance system, HEW's communication with WDG leaders and surveillance focal person of their supervising health facilities; 3) death review and response related characteristics such as WDG meeting at least monthly, monthly pregnant mothers’ conference, availability of steering committee’s meeting, frequency of meeting of the steering committee and time of verbal autopsy completion.

The questionnaire for health facilities (health centers and hospitals) (**S2 File**) also composed of three parts: 1) general information such as MPDSR committee meeting, availability of annual plan, multi-disciplinary MPDSR committee, monthly cluster review meeting, quarterly public conference, number of MPDSR trained members, profession and work experience of the MPDSR committee head 2) surveillance and surveillance related characteristics like: feedback mechanism, availability of updated pregnancy surveillance system, availability of line listing, availability of feedback from hospital, availability of report timeliness tracking mechanism and completeness of tracking mechanism 3) death review and response related characteristics such as time of facility based data abstraction completion, time of death reviewing after Verbal Autopsy (VA) and Facility Based Data Abstraction (FBDA), handling of the reviewed document and action planning status for the identified maternal and perinatal mortality.

After translating the questionnaires to local language (Tigrigna), then data were collected form the eligible participants at all levels, through 16 diploma nurse data collectors who were working in health facilities other than the study districts and have past experience of data collection, using face to face interview. Furthermore, document review was conducted to collect some data which were not possible to get form the individual participant interviews.

Three different open ended interview guides were prepared to conduct the qualitative interview with women development group, health facility and district health office managers and regional level expert. The interview guide for the FGD part was consisted of issues such as: functionality of the women development team on maternal and child health service utilization, contribution to early home delivery identification and awareness about MPDSR program (**S3 File**). Furthermore, the interview guide for health facilities and district health offices was addressed issues like: how they monitor the implementation of MPDSR, intersectoral collaboration on the program, challenges for the implementation of the program and actions taken and their recommendations on the way forward (**S4 File**). In addition to the issues inquired from health facilities and district health offices, a mechanism of program evaluation was also included in interview guide for the regional level manager (**S5 File**). Finally, data was collected from study participants using audio recording and supplementary field note by nine public health master holder professionals (two in each session) using the local language i.e. Tigrigna. Each interview was conducted in a convenient and confidential area and the duration of each interview was lasted from 1:30–2:00 hours for FGD and 1:00–2:00 hours for the in-depth interview.

### Measurement of variables

#### Early death identification

This variable was dichotomous variable (with a value of 1 = Yes, if the HEW identifies a death within 24hrs of the occurrence of the event and 0 = No, if the HEW failed to identify the event within 24hrs).

#### Quality of death notification

This variable was assessed in health posts for the formal death notification process (death notification in the recommended period of 24hrs, complete and proper death notification format utilization) with a value of “1 = Yes” for death notification within 24hrs of the occurrence of the event plus proper completion of notification format and “0 = No” for those which failed for either of the criteria).

#### Early death identification and notification

This dependent variable was obtained from thirteen different identification and notification related questions (timely maternal and perinatal death identification, availability of community perinatal death, way of maternal and perinatal death identification, availability of zero maternal and perinatal death report, proportion of zero maternal and perinatal death reports, availability of WDA’s weekly maternal and perinatal death reports, quality of maternal and perinatal death notification) from the health post’s questionnaire. Health posts above and below the mean scores were considered as practicing early death identification and notification with value of “1 = Yes” and “0 = No” respectively (**S1 Annex**).

#### Quality of death review

This dependent variable was derived from twenty death review related questions (availability of FBDA for maternal and perinatal death, availability of unmissed variable in maternal and perinatal FBDA formats, availability of maternal and perinatal VA formats from health posts, time of received maternal VAs, availability of unmissed variable in maternal and perinatal received VAs, availability of maternal and perinatal death reviews, availability of proper document handling, availability of formal maternal and perinatal death notification to district, availability of unmissed variable in maternal and perinatal death notification format, availability of maternal and perinatal death summary report, availability of unmissed variable in maternal and perinatal death summary reporting format) from the health facility based questionnaire. Health facilities above and below the mean score were considered as practicing good and poor quality of death review with values of “1 = Good” and “0 = Poor” respectively (**S2 Annex**).

#### Appropriate action

This dichotomous dependent variable was with a value of “1 = Yes “and “0 = No” for the appropriateness of the action taken following a death; for above and below mean scores respectively. It was computed from sixteen questions (availability of action plan for maternal and perinatal death, discussion with staff members and actions like: on job training, improvement of medical supply and equipment, availability/constructing of waiting maternity home, arrangement of layout of health facility, corrective measures like staff rotation, and discussions with WDA on issues like: on MPDSR program, importance of ANC (Antenatal Care), importance of skill delivery, importance of PNC (Postnatal Care), importance of birth preparedness and complication readiness, danger signs of pregnancy, newborn danger signs, and integration of MPDSR plan to the institution’s annual plan) from the health facility questionnaire (**S3 Annex**).

**Availability of timely Public Health Emergency Management (PHEM) report**: This variable was assessed from document of surveillance report; completed and timely reports of the year (2009 E.C) with a value of ‘1 = Yes’ and ‘2 = No’.

#### Uninterrupted pregnancy surveillance

In health post this was assessed using the pregnancy list registration book including this year (July-Dec. 2017) for its continuous and updated registering of mothers. In health centers, this was assessed using the availability of pregnant mothers’ list exchanging/referral linkage (including EDD (Expected Date of Delivery) notification) between the health center and health posts under its catchment. The values for this variable was ‘1 = Yes’ for the existence of uninterrupted pregnancy surveillance system and ‘2 = No’ for its absence.

### Operational definitions

#### Health facility

In this study this phrase was used for hospitals (primary, general and specialized) and health centers.

#### Community

In this study this word was used for selected health posts of kebeles.

### Data quality control measures

Data quality was ensured through two days training of data collectors on the objective of the study and data collection tools. In addition, pretesting of quantitative data collection tools was conducted and proper modification was made based on the feedback obtained during the pretest. Continuous supervision was also employed to ensure quality of quantitative data during data collection and entry. The quality of qualitative data was maintained by selecting participants who could gave more information like; women development team members and managers who are more exposed to the program. Additionally we took audio recorder and field notes simultaneously during the data collection period.

### Data management and analysis

For the quantitative study, data was entered to Epi-Info software version 7 and then transferred to SPSS version 21 for analysis and data cleaning and editing was done. Descriptive statistics was done to describe characteristics of participants and presented using frequency tables. The three outcome variables related to MPDSR analyzed in this study were: 1) early identification and notification, 2) quality of death review and 3) appropriateness of response.

Early identification and notification of maternal and perinatal death was analyzed from health posts’ data while quality of death review and appropriateness of the response were analyzed from health facilities’ data.

Odds ratio with 95% confidence interval was calculated to determine the strength of association of selected independent variables with the outcome variables. Variables with p-value of <0.25 at bi-variate analysis were further analyzed using multivariable logistic regression models to identify the independent predictors of the outcome variables and finally significant association was declared at p-value of < 0.05.

The first five authors and four supervisors transcribed and translated the qualitative data with reference to the Tigrigna audio recordings for clarification. The coding framework followed the topic guide and texts were coded and eight categories (institutionalization, community participation, intersectoral collaboration, communication, community awareness, capacity of health work force, logistic, equipment and infrastructure and system strengthening) were developed which later merged into three broad themes (implementation, challenges and prospects). These themes were settled before analysis was started by the first five authors. Then the first author (BA) conducted the analysis manually using thematic content analysis following data familiarization (reading and re-reading of the transcripts) on translated English transcripts and refinement was made through discussion with all authors.

### Ethical consideration

Ethical clearance was obtained from the ethical review committee of Tigray Health Research Institute (THRI) with ref number of THRIRM/0036/2010. Official permission letter was obtained from Tigray Regional Health Bureau (TRHB) and selected districts. The respondents were informed about the objective and purpose of the study and written consent was obtained. However; for the focused group discussion participants, the data collectors’ signature, which was signed after explaining the objective, was considered to record/document the consent of the participants. Confidentiality of the information was assured and participants were informed that they have the right to withdraw from interview at any stage of the interview.

## Results

### Quantitative findings

In the quantitative part of the study, 75 health posts and 66 health facilities (1 referral, 10 general and 5 primary hospitals and 50 primary health care units) from 22 districts were included.

#### Identification and notification of maternal and perinatal death in health posts

Sixty-four (87.7%) of health posts identify maternal death within 24 hours, but 60 (82.2%) of health posts did not formally report maternal death to the next higher level within 24 hours. In this study 34 (45.3%) health posts practice early identification and notification of maternal and/or perinatal death. Only 10 (13.3%) of health posts reported the presence of perinatal death in their Kebele and 9 (69.2%) of health posts conducted verbal autopsy (VA) following formally reported maternal death (**Table 2**).

**Table 2:** Maternal and perinatal death identification and notification activities in the implementation of MPDSR at health posts, Tigray, Northern Ethiopia, 2018 (*N = 75).

Variable	Frequency	(%)
**Identification of maternal death**	** **	** **
Within 24hrs	64	87.7
> 24hrs	9	12.3
**Health posts formal maternal death notification**		
Yes	13	17.8
No	60	82.2
**Time of maternal death notification by Health post’s**		
Within 24 hrs.	9	69.2
> 24hrs	4	30.8
**Availability of missed variable in the MDN format**		
Yes	8	61.5
No	5	38.5
**Health post’s quality of death notification for maternal death**		
Good	5	38.5
Poor	8	61.5
**Presence of community perinatal death in 2009**		
Yes	10	13.3
No	65	86.7
**Identification of perinatal death**		
Within 24hrs	4	40
> 24hrs	6	60
**Over all practice of early identification and notification**		
Yes	34	45.3
No	41	54.7
**Quality of formal notification for perinatal death**		
Good	1	10
Poor	9	90
**Verbal autopsy for maternal death done**		
Yes	9	69.2
No	4	30.8
**Time of VA for maternal death done**		
Within four weeks	6	66.7
>four weeks	3	33.3
**Availability of missed variable in the maternal VA format**		
Yes	3	33.3
No	6	66.7
**Participation of HEWs in MPDSR death review**		
Yes	62	82.7
No	13	17.3

* N may not be necessary 75 as two health posts were assessed due to perinatal death

MDN-Maternal Death Notification, VA- Verbal Autopsy

#### Death identification and notification in health facilities

Twenty-four (48.0%) and 3 (6.0%) of the assessed primary health care units reported community based maternal and perinatal deaths respectively. On the other hand, 59 (70.2%) of maternal and 1291 (99.3%) perinatal deaths were reported from health facility based maternal and perinatal deaths reporting system while 25(29.8%) maternal and 9(0.7%) perinatal deaths occurred at community. Furthermore, only one third 9(36%) and 32(88.9%) of health posts notified maternal death formally and informally where very few of them were notified within 24 hours maternal death (**Table 3)**.

**Table 3:** Maternal and perinatal death identification and notification related activities in the implementation of MPDSR in health facility, Tigray, Northern Ethiopia, 2018. (**N = 66).

variables	Maternal Death	Perinatal Death
	Number (%)	Number (%)
**PHCUs with community death**		
Yes	24(48.0)	3(6.0)
No	26(52.0)	47(94.0)
**Numbers of death**		
At community	25(29.8)	9 (0.7)
At health facility	59 (70.2)	1291 (99.3)
**Informal death notification by HPs**		
Yes	32(88.9)	2(66.7)
No	4(11.1)	1(33.3)
**Formal death notification by HPs**		
Yes	9(36)	0
No	16(64)	3(100)
**Time of formally death notification by HPs**		
Within 24 hours	5(55.6)	NA
Greater than 24 hours	4(44.4)	NA
**Formal death notification to head of HF by HFs**		
Yes	12(29.3)	4(8.5)
No	29(70.7)	43(91.5)
**Numbers of death formally notified**		
Yes	20(33.9)	24(1.9)
No	39(66.1)	1267(98.1)
**Time of formally death notification**		
Within 24 hours	20(100)	24(100)
Greater than 24 hours	0	0
**Health facilities’ without missed variables in the notification formats**		
Yes	7(58.3)	2(50)
No	5(41.7)	2(50)
**Numbers of notification formats without missed variables**		
Yes	13(65)	18(75)
No	7(35)	6(25)
**Availability of weekly death report(including zero report)**		
Yes	47(71.2)	6(9.1)
No	19(28.8)	60 (91.9)

**N may not be necessary 66 as the table consists performance of the health facilities.

HF-Health Facility HPs-Health Posts

#### Death review in health facilities

Half of the health facilities took Facility Based Data Abstraction (FBDA) for maternal death whereas only 8(17%) health facilities took FBDA for perinatal death. Thirty two (48.5%) of health facilities received verbal autopsy for maternal death from community while no health facility was received verbal autopsy for perinatal death. Furthermore, only 29 (43.9%) and 7 (14.9%) of health facilities sent maternal and perinatal death summary reports respectively. In this study, 36 (54.5%) health facilities were practicing good quality of death review following maternal and/or perinatal deaths (**Table 4**).

**Table 4:** Maternal and perinatal death review related activities in the implementation of MPDSR in health facility, Tigray, Northern Ethiopia, 2018. (**N = 66).

variables	Maternal death	Perinatal death
	Number (%)	Number (%)
**Health facility conducted FBDA**		
Yes	18(50)	8(17)
No	18(50)	39(83)
**Numbers of deaths with FBDA**		
Yes	36(61)	60(4.6)
No	23(39)	1231(94.4)
**Timing of FBDA conducted**		
Within 24 hours	12(33.3)	31(51.7)
From 24 hours to 1 week	19(52.8)	12(20)
Greater than 1 week	5(13.9)	17(28.3)
**Health facilities’ without missed variables in the FBDA formats**		
Yes	8(44.4)	6(75)
No	10(55.6)	2(25)
**Numbers of FBDA formats without missed variables**		
Yes	22(61.1)	32(53.3)
No	14(38.9)	28(46.7)
**Health facility received VA**		
Yes	32(48.5)	0
No	23(34.8)	0
**Numbers of deaths with VA and received in health facility**		
Yes	37(44)	0
No	47(56)	3(100)
**Timing of VA received**		
Within 3–4 weeks	31(83.8)	0
Greater than 4 weeks	6(16.2)	0
**Health facilities’ without missed variables in the VA formats**		
Yes	9(13.6)	0
No	23(34.8)	0
**Health facilities conducted death review**		
Yes	39(59.1)	4(8.5)
No	27(40.9)	43(91.5)
**Numbers of reviewed deaths**		
Yes	55(65.5)	8(0.6)
No	29(34.5)	1292(99.4)
**Timing of death reviewed**		
With one week	42(76.4)	3(37.5)
Greater than one week	13(23.6)	5(62.5)
**Health facilities send death summary report**		
Yes	29(43.9)	7(14.9)
No	37(56.1)	40(85.1)
**Overall quality of death review**		
Good	36 (54.5)	
Poor	30 (45.5)	

**N may not be necessary 66 as the table consists performance of the health facilities.

#### Health facility’s response to maternal death

As a response to prevent further maternal and perinatal death, one third 22(33.3%) of the health facilities developed at least one action plan to respond for maternal death but only one health facility has an action plan to respond for perinatal death. Among the health facilities, only 35(53%) of them took proper action/response following the occurrence of death. Proper action to maternal and perinatal death include, discussion with staff following maternal death 46 (66.7%) and health facilities made discussion with Women Development Group (WDG) 40(72.7%) (**Table 5**).

**Table 5:** Maternal and perinatal Death response related activities in the implementation of MPDSR in health facilities, Tigray, Northern Ethiopia, 2018 (**N = 66).

variables	Number	(%)
**Health facility developed action plan after maternal death review**		
Yes	22	33.3
No	44	66.7
**Health facility developed action plan after perinatal death review**		
Yes	1	2.1
No	46	97.9
**Numbers of action plan developed for maternal death**		
Yes	28	33.3
No	56	66.7
**Numbers of action plan developed for perinatal death**		
Yes	1	0.1
No	1299	99.9
**Health facility made discussion with staff regarding death response**		
Yes	46	66.7
No	20	30.3
**Health facility made discussion with WDAs regarding death response**		
Yes	40	72.7
No	15	27.3
**Over all proper action/Response taken**		
Yes	35	53.0
No	31	47.0

**N may not be necessary 66 as the table consists performance of the health facilities.

#### Factors associated with MPDSR implementation

Three outcomes of the MPDSR implementation were examined in the current study. The first outcome was availability of early identification and notification of MPDSR in health posts. Hence, the factors that were associated with early identification and notification of MPDSR in bivariate logistic regression were number of health extension workers in the Kebele, involvement of Kebele chairman in maternal death review and steering committees participation. Kebeles with 3–4 health extension workers were three times more likely to early identify and notify maternal and/or perinatal death (MPD) in health posts. In addition, health posts which involve Kebele administration were four times more likely to early identify and notify MPD than those that did not involve. In addition, health post which involve the steering committee are four times more likely to early identify and notify MPD than those who did not involve.

In multivariate logistic regression analysis, number of health extension workers in the Kebeles, availability of PHEM reports and steering committees involvement were associated with early identification and notification of MPD. Kebeles with 3–4 health extension workers are six times more likely to early identify and report MPD than those with lesser number of health extension workers. Availability of PHEM report were also four times more likely to early identify and notify maternal death than their counter parts. Health posts which involve steering committee were nine times more likely to early identify and notify MPD than those that did not involve (**Table 6**).

**Table 6:** Logistic regression analysis of variables associated with early identification and notification of MPDSR in health posts, Tigray, Northern Ethiopia, 2018 (N = 75).

Variables	EIN		COR (95%CI)	AOR (95%CI)
	Yes: n (%)	No: n (%)		
**Population in the catchment health post**				
≤ 5000	7 (30.4)	16 (69.6)	0.34 (1.00,1.13)	0.28 (0.05,1.39)
5001–7500	14 (48.3)	15 (51.7)	0.72 (0.24,2.16)	0.95 (0.23,3.91)
≥ 7501	13 (65.5)	10 (43.5)	1	1
**Number of HEWs in the Kebele**				
1–2	15 (34.1)	29 (65.9)	1	1
3–4	19 (61.3)	12 (38.7)	3.06 (1.18,7.95) *	**6.09 (1.51,24.49)** *
**HP having HEWs all with diploma and above**			1	1
Yes	8 (34.8)	15 (65.2)	0.53 (0.19,1.47)	0.38 (0.10,1.50)
No	26 (50.0)	26 (50.0)		
**Participation of Kebele admin in MDR**				
Yes	29 (54.7)	24 (45.3)	4.11 (1.32,12.77) *	2.26 (0.53,9.61)
No	5 (22.7)	17 (77.3)	1	1
**Availability of timely PHEM repots**				
Yes	26 (52.0)	24 (48.0)	2.30 (0.84,6.30)	**4.39 (1.08,17.80)** *
No	8 (32.0)	17 (68.0)	1	1
**Availability of un-interrupted pregnancy surveillance**				
Yes	26 (50.0)	26 (50.0)	1.86 (0.68,5.18)	1.79 (0.50,6.48)
No	8 (34.8)	15 (65.2)	1	1
**Steering committee’s participation**				
Yes	17(68.0)	8(32.0)	4.13 (1.48,11.49) *	**9.19 (1.31,64.34)** *
No	17(34.0)	33(66.0)	1	1
**HEW’s discussion with WDA about cause of death**				
Yes	18 (56.3)	14 (43.8)	2.17 (0.85,5.52)	0.52 (0.10,2.83)
No	16 (37.2)	27 (62.8)	1	1

EIN- Early identification and notification, MDR-maternal death review

* Significant at p-value of < 0.05.

The second outcome variable was quality of MPDSR where it is dichotomized as good or poor. Accordingly, the factors that were associated with good quality MPDSR in the bivariate analysis were availability of MPDSR trained nurse, work experience of the HF, PHEM focal person trained for MPDSR, MPDSR incorporated as annual plan. Health facilities with at least one MPDSR trained nurse were five times more likely to have good quality MPDSR. Health facilities headed by a person with more than 7 years’ work experience were three times more likely to have good quality MPDSR than those headed with lesser experience. PHEM trained as focal person for MPDSR is four times more likely to have good quality MPDSR than those who have not. In addition, Health posts which included MPDSR as their annual plan were three times more likely to report good quality MPDSR than those who did not have an annual plan.

In a multivariate analysis, availability of at least one MPDSR trained nurse and experiences of the head of the health facility were associated with good quality MPDSR. Health facilities with at least one trained nurse were three times more likely to have good quality of MPDSR and health facilities headed by a person who have greater than seven years’ experience were more likely to have good quality of MPDSR than those headed with lesser experience (**Table 7**).

**Table 7:** Logistic regression analysis of variables associated with quality of death review of MPDRS in health facilities, Tigray, Northern Ethiopia, 2018 (N = 66).

Variables	Quality of death review		COR (95%CI)	AOR (95%CI)
	Good: n (%)	Poor: n (%)		
**Availability of at least one nurse trained for MPDSR**				
Yes	26 (72.2)	10 (27.8)	**5.20 (1.82,14.90)** *	**3.75 (1.08,12.99)** *
No	10 (33.3)	20 (66.7)	1	1
**Health facility’s head trained for MPDSR**				
Yes	15 (65.2)	8 (34.8)	1.96 (0.69,5.59)	0.90 (0.24,3.39)
No	21 (48.8)	22 (51.2)	1	1
**HF's head work experience**				
≤6 years	10 (37.0)	17 (63.0)	1	1
≥ 7 years	26 (66.7)	13 (33.3)	**3.40 (1.22,9.50)** *	**3.70 (1.04,13.22)** *
**PHEM focal person trained for MPDSR**				
Yes	28 (65.1)	15 (34.9)	**3.50 (1.21,10.13)** *	1.94 (0.49,7.77)
No	8 (34.8)	15 (65.2)	1	1
**MPDSR incorporated as part of annual plan**				
Yes	24 (66.7)	12 (33.3)	**3.00 (1.10,8.21)** *	2.54 (0.79,8.10)
No	12 (40.0)	18 (60.0)	1	1
**Proportion of sent weekly PHEM reports**				
≥ 85%	24 (64.9)	13 (35.1)	2.62 (0.96,7.12)	1.86 (0.56,6.24)
< 85%	12 (41.4)	17 (57.6)	1	1

*Significant at p-value of < 0.05.

The third outcome variable was proper action implementation of MPDSR as categorized as proper and improper action implementation. Accordingly, in bivariate analysis only health facilities with at least one cluster review meeting were four times more likely to have proper action implementation than those with no cluster review meeting. In multivariate logistic regression, health facilities with at least one cluster meeting and health facilities with uninterrupted pregnancy surveillance were associated with proper action implementation. Health facilities with at least one cluster review meeting were five times more likely to properly implement MPDSR action. In addition, health facilities which conduct uninterrupted pregnancy surveillance were seven times more likely to properly implement MPDSR action (**Table 8**).

**Table 8:** Logistic regression analysis of variables associated with proper action implementation of MPDSR in health facilities, Tigray, Northern Ethiopia, 2018 (N = 66).

Variables	Took proper action		COR (95%CI)	AOR (95%CI)
	Yes: n (%)	No: n (%)		
**HFs with at least one nurse trained for MPDSR**				
Yes	22 (61.1)	14 (38.9)	2.06 (0.77,5.50)	2.05 (0.58,7.23)
No	13 (43.3)	17 (56.7)	1	1
**HFs with at least one HO trained for MPNDSR**				
Yes	14 (66.7)	7 (33.3)	2.29 (0.78,6.73)	3.75 (0.74,18.97)
No	21 (46.7)	24 (53.3)	1	1
**HFs with head trained for MPNDSR**				
Yes	15 (65.2)	8 (34.8)	2.16 (0.76,6.14)	0.96 (0.20,4.60)
No	20 (46.5)	23 (53.5)	1	1
**HF's head work experience**				
Yes	12 (44.4)	15 (55.6)	1	1
No	23 (59.0)	16 (41.0)	1.80 (0.67,4.84)	0.89 (0.24,3.34)
**Duration of chairman leading the MPDSR:**				
One year and less	13 (39.4)	20 (60.6)	1	1
Above one year	22 (66.7)	11 (33.3)	3.08 (1.13,8.41)	3.15 (0.90,10.99)
**HFs with at least one cluster review meeting**				
Yes	18 (75.0)	6 (25.0)	4.41(1.45,13.39) *	**4.87(1.30,18.26)** *
No	17 (40.5)	25 (59.5)	1	1
**HFs conducted public conference with MPNDSR agenda**				
Yes	15 (68.2)	7 (31.8)	2.57 (0.88,7.54)	1.92 (0.49,7.47)
No	20 (45.5)	24 (54.5)	1	1
**Availability of important formats in the HF**				
Fair	12 (70.6)	5 (29.4)	2.71 (0.83,8.87)	2.54 (0.62,10.38)
Poor	23 (46.9)	26 (53.1)	1	1
**HFs uninterrupted pregnancy surveillance**				
Yes	27 (67.5)	13 (32.5)	3.12 (0.91,10.62)	**6.85(1.22,38.54)** *
No	6 (40.0)	9 (60.0)	1	1

*Significant at p-value of < 0.05.

### Qualitative findings

We asked 11 key informants (three district health office head/deputy head, three PHEM/MCH experts, four PHCU directors and one regional expert) and 3 focused group discussions (6–8 members of WDG each) to collect data for the qualitative study with the principle of saturation level. Twenty five women development group leaders were included in the three FGD and most of them were farmers. In the in-depth interview except the regional MPDSR focal person all of them were male participants with educational level of bachelor degree.

#### Implementation

**Institutionalization**

According to most of the in-depth interview participants, though maternal death identification and notification was the same in all districts, the reviewing process (starting from completion of: verbal autopsy to proper maternal death reporting format utilization) was not the same; in some districts most of these activities were conducted by PHCU level and by district health offices in other districts. Furthermore, there was no clear understanding about the newly developed Ethiopian national MPDSR guide line among district health office experts for the reason that most of them were not trained for the updated national guideline while there was no major difference between the pre-prepared regional guideline and updated national guideline. The updated national guideline allows decentralization of death review process to primary health care units. Additionally, experts of district health offices’ were reflected their fear on challenges of effective community mobilization in a district based manner. However, they emphasized on the importance of death review by health facilities which facilitated more death reviews. Additionally, all participants acknowledged the importance of MPDSR in reducing maternal and perinatal death.

“*Though decentralization is important*, *we have a fear that PHCU’s committee may give response to their catchment population but could not to the whole district’s community” District health office MCH expert*.

**Community participation**

Most of the in depth interview participants agreed that most of the maternal deaths were identified and reported timely with an active involvement of the community members especially women development team members. However, due to fear of legal repercussion some community members ignore reporting deaths occurred in their area.

“*Though majority of the community members are volunteer to report deaths occurred in the community*, *some of them also hide maternal deaths for fear of legal accountability*.*”(District* health office *MCH expert)*.

Participants were agreed on the importance of active involvement of the community as a response following maternal death like; discussions and health educations to improve the awareness of the community. However, some community members were not considered these activities as responsive activities especially if deaths are occurred in health facilities. Study participants from FGD were reflected that nothing is to be done following health facility based maternal deaths other than mourning. FGD participants were complained health facilities’ death with the negligence of health facilities to share the feedback on the cause of the maternal deaths which made them to perceive dissatisfied by the service given in the health facilities.

“*If the death is happened*, *there is no other effort done at community level even with health professionals without crying” (FGD participant)*.

**Intersectoral collaboration**

Most participants acknowledged the importance of intersectoral collaboration with different sectors like: women association, women’s affaire, education and agriculture, during conducting maternal death review so as to take measurable actions as a response in collaborative fashion. However, involvement of different sectors during review could create a fear of legal repercussion among the professionals and could results in a lower rate of death review and hinders the implementation of the next step i.e. response part of MPDSR implementation.

“*Conducting death review with different sectors helps us in acceptability and trustworthy by different bodies like of the community and other organizations” (MCH expert from district health office)*.

**Communication**

Most of the participants from the in-depth interview complained that hospitals were not voluntary to give feedback of the death review for cause of maternal death which became a challenge in taking appropriate actions so as to prevent further deaths.

“*If hospital mangers and staff members are not supportive*, *death reviewing is time consuming and it is not possible to take appropriate actions*: *like clarifying the community*, *which help in solving the fear of the community to utilize service and preventing further maternal death*” *District health office MCH expert*.

Furthermore, FGD participants were reflected that they did not inform on the cause of maternal deaths occurred in hospitals. They also indicate this created a negative perception for future and further health service utilization by the community.

“*We do not know still the cause of maternal death and no action was done after the death of pregnant women even by health professionals here in the health center*” FGD participant’s response related to maternal death happen in hospital before one year of the data collection. Additionally participants of the in-depth interview acknowledged the importance of horizontal feedback communication among health facilities with the same level.

“*Giving feedback is not only for hospitals to down step; but also*, *for health centers which give service for mothers out of their catchments either in the same district or in different districts*. *Horizontal and vertical information sharing is critical to avert maternal death” PHCU director*.

#### Challenges

**Community awareness**

Community awareness on the report-ability of maternal death was reported to be good. Maternal death issue is becoming more sensitive issue in the community. However; perinatal deaths identification and reporting were not paid a due focus both by the health professionals and community members. Some participants from the focus group discussions reflect that they have no awareness about report-ability of perinatal death.

“*There is no other efforts done and we are not informed what to do after the occurrence of perinatal death” (FGD participant)*.

Community awareness on the cause of maternal death and knowledge on danger signs during pregnancy and post-partum period were limited according to the reflections of participants of the in-depth interview. Bad traditional thinking like; “Ganien Hizwa” or “Buda Hizwa” to mean “bedevil” or “evil eye” were reported both in urban and rural dwellers.

According to FGD participants, gaps were observed from both community and the service providers. The community had wrong perception that no death could occurred in health facilities. On the other hand health professionals did not work fully to aware the community about the delay models as causes of maternal death.

“*The pregnant woman died in 2009 E*.*C was already referred and follow at the health center and the responsibility is the health professionals who are working there*. *Rather than referring to the health facility*, *no other efforts were done after the maternal death” FGD participant*.

**Capacity and capacity building**

Most in-depth interviews raised limited human workforce capacity as a challenge to MPDSR implementation successfully. They reflected that some community members lose confidence on the service they received from health facilities. Failure to identify risks early, immediate home delivery after seen by health professionals and rude character of service providers were some of the mentioned factors that negatively affected the future health service utilization which were reflected both by in-depth and FGD participants. For their bad experience in health facilities some mothers preferred home to health facility delivery. For instance a mother who experienced neonatal death due to delayed referral from health center to hospital was delivered at home for her recent delivery though she had had ANC visit.

“*When I follow pregnant mothers in my team*, *I advise them to utilize health services like ANC and facility delivery while I myself did not do it during my second delivery” (FGD participant)*.

Furthermore, not receiving training on the newly updated MPDSR national guide line for district health office experts and high turnover of trained staff members in health facilities were the commonly explained capacity related challenges for smooth implementation of MPDSR.

“*Experts in districts should be capacitated*, *if they are expected to support and help health centers in any activities which realized implementation of a program successfully” (district health office PHEM expert)*.

**Logistic, equipment and infrastructure**:

Lack of standardized formats including the national guideline and infection prevention materials like autoclave were explained as some challenges for proper implementation of the recommended actions for response. Furthermore, lack of maternity waiting homes, shortage and unequipped neonatal intensive care units, shortage of ambulances and absence of year round roads with long distance were the infrastructure related factors hindering the action for further maternal and perinatal death prevention.

“*We are mobilizing the mothers to wait in the health facilities; however we do not have rooms to accommodate them and this creates a problem on trustworthiness for the mobilization we give” MCH district health office expert*.

#### Prospects

**Systems strengthening**

Program based supervisions up-down level, giving capacity building trainings in an integrative way to health care providers (including the district health office experts), timely integration of perinatal death reporting space in the PHEM weekly reporting format, improving the infrastructure of health facilities like road access to each Kebele and building expansion, strengthening referral feedback exchange among facilities and special community mobilization to increase the awareness on perinatal death were the most recommended area of focus for highlighting the way for successful implementation of MPDSR.

“*We health professionals and the community as a whole are sensitive to maternal death but not neonatal death especially in the community*. *Therefore it is important to give awareness creation mobilizations to the community” district health office PHEM expert*.

“*It is very important to expand maternity waiting homes in health facilities and actors (partners) should take the responsibility and regional health bureau should mobilize/search partners to do so” district health office MCH expert*.

## Discussion

This study aims to examine the policy practice gap in the implementation of MPDSR in Tigray region, Ethiopia involving community and health facility-based practice.

In this study, only 34 (45.3%) of health posts were practicing early identification and notification of maternal/perinatal death. Furthermore, only 36 (54.5%) and 35 (53%) of health facilities were practicing good quality of death review and took proper action following maternal and/or perinatal deaths respectively.

This study revealed that 64 (87.7%) and 4(40%) of the assessed health posts with maternal and community perinatal death were identifying the occurrence of deaths within 24 hours; among which only 13 (17.8%) and none of them notify formally for maternal and community perinatal deaths respectively. Maternal/perinatal death identification and notification was low compared with national target of 95% [31]. According to the qualitative finding, inconsistency in implementation was reported which could contributed for this low finding. The identification and notification formats were found either in health centers or district health offices which could be an indication for differently implementation. On the other hand, the timely maternal death identification was fair. This may be related to the active participation of community members mainly women development groups as demonstrated in the qualitative findings. Furthermore, the qualitative findings also revealed that maternal death issue is more pronounced and considered as a big loss than perinatal death which could result in good performance of maternal death identification.

Death review requires investigating all maternal and perinatal deaths reported from community and health facilities to identify contributing factors [32, 33]. However; in this study only 55 (65.5%) and 8 (0.6%) of maternal and perinatal deaths were reviewed respectively. This finding (the maternal part) is higher than other studies from Kenya and Guinea that reported review of half of maternal deaths [34, 35]. In contrast, this finding is lower compared to the national target of 90% [31] and the finding from Uganda which reported review of 71% of maternal death and 33.3% of perinatal deaths [36]. Furthermore, the qualitative finding revealed that unnecessary delay and poor feedback to down step was observed in some health facilities which might contribute to the variations between findings of the current and other studies.

Intersectoral collaboration during death review (for maternal death) was indicated as important factor for response. On the other hand, it was also indicated that this could lead to low deaths review due to fear of legal repercussion issue [28, 37]. The qualitative finding highlighted that some health facilities delay in the death review process and resist giving feedback for those who want to know the cause of death. This may be due to poor practice and awareness of “no name, no shame” principle of MPDSR from the triple side i.e. the reviewers, service providers and service receivers. This could affect the timely reviewing process and sharing feedbacks among concerned bodies and thus affect the commencement of timely response [36, 38].

The primary purpose of MPDSR is response based on information obtained from the death review to prevent such deaths in the future [28, 32]. According to the national technical guideline for MPDSR all health facilities should develop action plans maternal or perinatal death (100%) [31]. However, in this study, only one-third 22 (33.3%) and only 1 (2.7%) of the health facilities develop at least one action plan for maternal and perinatal deaths respectively. This is also lower than the findings from Zimbabwe which revealed 69% health facilities developed action plans with recommendations after maternal death [39]. Poor document handling may contribute to the low level of development of action plan in this study.

In this study perinatal death identification, notification, death review and response were neglected. Similarly result from systematic review in low- and middle-income countries highlighted that perinatal deaths are more neglected than maternal death though women and their babies share the same period of highest risk [39]. Qualitative finding of the current study also indicated poor community awareness. Furthermore, events happened in health facilities were not reported and reviewed. These findings are similar with the findings of other studies [40, 41] which revealed underestimated reports for perinatal deaths. This could be exacerbated by poor community awareness [40], high perinatal death occurrence in HFs [36] and poor knowledge of health professionals [41]. Moreover, from our observation, the PHEM reporting format in the region did not include a space for perinatal death, which could create reluctance in all levels.

Three to four numbers of HEWs was one of the significant variables for early identification and notification of maternal and perinatal deaths in health posts. This may be due to the reason that in kebeles with high number of HEWs, it reduce the individual burden by distributing the population as they may share each “Gotts/Kushets” (villages), including the hard to reach areas, among themselves; and this could create an opportunity to detect events timely [42, 43].

Steering committee’s participation in death response was also the other predictor variable for early death identification and notification. Their active participation in death response activities could alert women development group members so as to take action to prevent further deaths and report events timely/early. This finding is supported by different studies which revealed active participation of community members could increase the information sharing and to take important measures [44–46].

Furthermore, availability of timely PHEM report was also another significant factor for early death identification and notification. Health posts which had timely PHEM reports were more likely to practice early death identification and notification when compared with their counterparts. This may be due to the reason that: those who had timely report could practice active surveillance which could help them in detecting newly events in their catchments using different methods to get information from the community members including the steering committee members [45].

Experience of HF’s head was one of the influencing variables for quality of death review in health facilities. More years of experience was significantly associated with good quality of death review. This may be due to the reason that more years of experience could expose the head with different capacity building trainings, awareness creation review meetings [36] and improvements of skills due to learning by doing [39]. Additionally from the qualitative part of the study turnover health providers was explained as one of the challenges for smooth implementation of MPDSR.

Furthermore, availability of at least one trained nurse was also the other significant variable for quality of death review. This may be due to; presence trained person could create an opportunity to share responsibilities among the trained one, focal person and head of the facility which could result in good quality of death review process. This is supported by other findings which revealed workload could hinder the smooth implementation of MPDSR[36, 39].

Conducting at least one cluster review meeting was an independent significant contributor for proper action implementation following maternal or perinatal death. This may be due to the fact that such review meetings could make participants to take responsibility so as to prevent further deaths [36]. Additionally, uninterrupted pregnancy surveillance was the other variable that significantly affects the proper action implementation. This could be due to the reason that: if pregnancy surveillance is active, every stakeholder like; women development group (WDG) and steering committee could be active to attend the pregnancy outcome of each registered pregnant mothers. This finding is supported by a study from Pakistan which revealed that higher mortality detection by enhanced pregnancy surveillance system [47].

### Strength and limitation

This study is the first study which employed mixed method after the implementation of the program in Ethiopia. Therefore, its finding could critically important for countries to strengthen the implementation of MPDSR. However; as the study considered previous one year performances recall bias may be introduced and poor document handling may also affect the finding. Furthermore, community death may be underestimated due to report hiding.

## Conclusion

The practice of early identification and notification was found low in health posts in this study. Furthermore, the proportions of health facilities that practiced good quality of death review and took proper action following the occurrence of death were low.

Community participation, number of HEWs, active surveillance including pregnancy surveillance, review meetings and capacity of health work force were some of the significant factors influencing the implementation of MPDSR. Therefore, strengthening active surveillance with active community participation alongside with strengthening capacity building trainings and employing additional HEWs with special focus to improve the awareness could enhance the implementation of MPDSR.

## Supporting information

S1 Fig S1 Fig which was used as schematic presentation of sampling producer. (TIF)

S1 File This is the S1 File questionnaire which was used to collect a data from health posts. (DOCX)

S2 File This is the S2 File questionnaire which was used to collect a data from health facility. (DOCX)

S3 File This is the S3 File questionnaire which was used to collect a qualitative data from FGD. (DOCX)

S4 File This is the S4 File questionnaire which was used to collect a qualitative data from health facilities and district health offices. (DOCX)

S5 File This is the S5 File questionnaire which was used to collect a qualitative data for the Regional Health Bureau expert. (DOCX)

S1 Annex This annex contains the variables which were used to compute the early maternal/ perinatal death identification and notification. (DOCX)

S2 Annex This annex contains the variables which were used to compute quality of death review in MPDSR implementation. (DOCX)

S3 Annex This annex contains the variables which were used to compute appropriate action In MPDSR implementation. (DOCX)

S1 Appendex This appendex contains the STROBE check list of the manuscript. (DOC)

## Abbreviations

FBDA: Facility Based Data Abstractuion
FGD: Focus Group Discussion
HEWs: Health Extension Workers
HFs: Health Facilities
KIIs: Key Informant Interviw
MCH: Maternal and Child Health
MDSR: Maternal Death Surveillance and Response
MPDSR: Maternal and Perinatal Death Surveillance and Response
MPD: Maternal and/or Perinatal Death
MMR: Maternal Mortality Ratio
NMR: Neonatal Mortality Ratio
PHCU: Primary Health Care Unit
PHEM: Public Health Emergency Management
VA: Verbal Authopsy
WDA: Women Development Group, hrs-hours
