# Supplementary figures and images for: Maternal and perinatal death surveillance and response in Ethiopia: Achievements, challenges and prospects
Source: PLoS One. 2019 Oct 11;14(10):e0223540. doi: 10.1371/journal.pone.0223540 (PMC6788713; doi:10.1371/journal.pone.0223540)

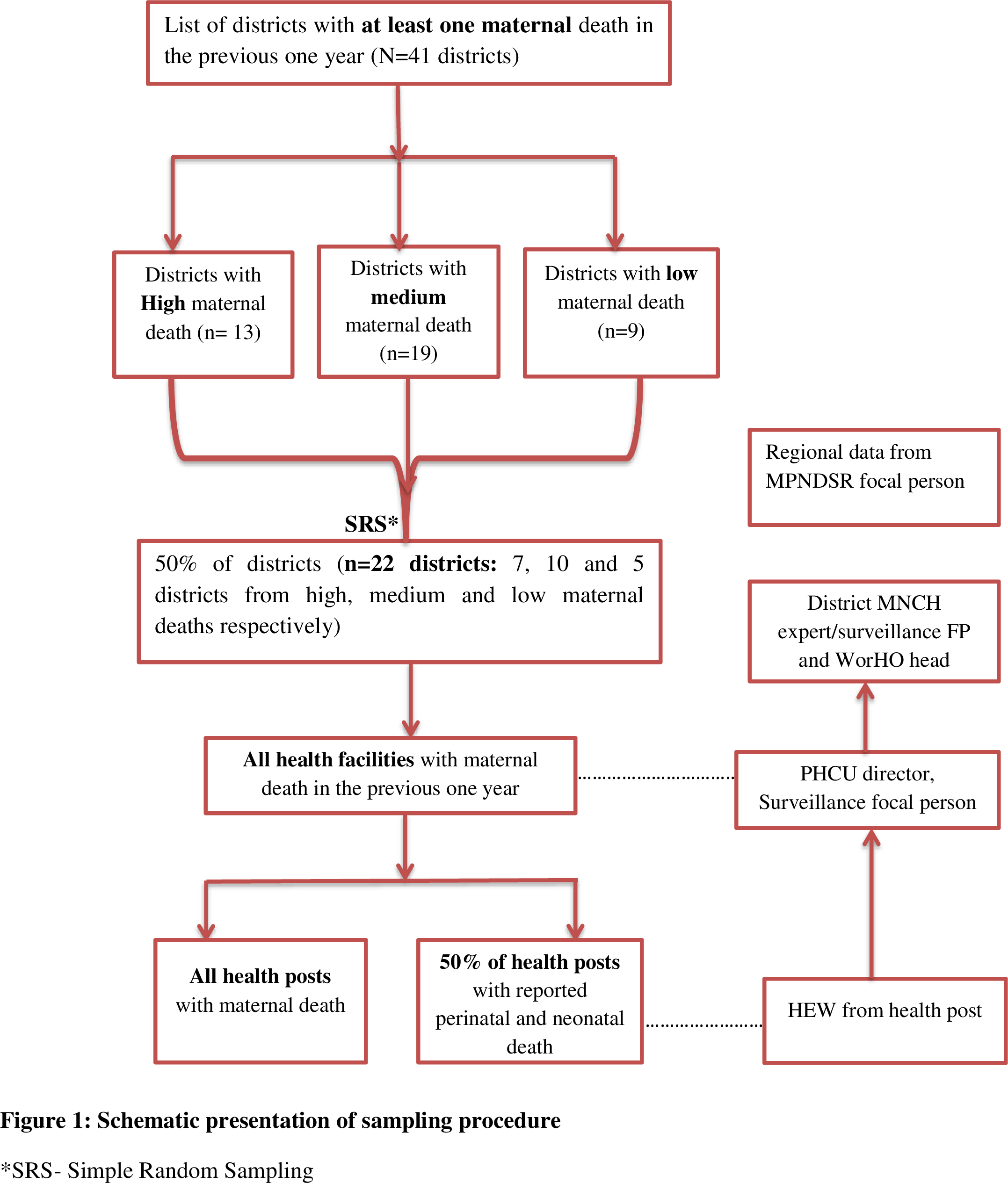

Supplement: S1 Fig — (TIF) [file pone.0223540.s001.tif]
