# Supplementary material for: Maternal and perinatal death surveillance and response in Ethiopia: Achievements, challenges and prospects
Source: PLoS One. 2019 Oct 11;14(10):e0223540. doi: 10.1371/journal.pone.0223540 (PMC6788713; doi:10.1371/journal.pone.0223540)
Supplement: S1 File — (DOCX) [file pone.0223540.s002.docx]

**Health Post Questionnaire**

**Inform sheet and consent form**

**Introduction**

My name is ---------------------------------I am a member a research team conducting by Tigray Health Research Institute in collaboration with Tigray Regional Health Bureau and UNFPA on **Implementation Status of Maternal, Perinatal and Neonatal Death Surveillance and Response in Tigray Region, Northern Ethiopia**. This study is commencing in health facilities of selected 22 districts of Tigray and your health facility/organization is among these facilities. We are interviewing head of health facilities, HEWs and PHEM focal persons to obtain the information. Your involvement in this research is important to know the implementation status of MPNDSR programme in the region. The questionnaire will take around hour and your name will not be written in this form and will never be used in connection with any of the information you tell me. You do not have to answer any question that you do not want to and you may end this interview at any time you want to. There is no risk for being not participating in the study. However, your participation will help us to know the implementation status of MPNDSR programme. We appreciate your help in responding to this Research questions.

Do have willingness to participate?

No----------- (Say “Thank you!!” and stop here!)

Yes---------- (Say “Thank you!!” and make sure that you got the signature of the participant)

Respondent’s signature-------------------------------------------------------- date----------------

Responsibility of the participant-----------------------------

Name of interviewer ----------------------------- signature-------------------- date----------------

Questionnaire code ------------------

Name of supervisor ------------------------ signature---------------- date----------------

**Tigray Health Research Institute**

**Implementation Status of Maternal, Perinatal and Neonatal Death Surveillance and Response in Tigray Region, Northern Ethiopia**

# Annex I: Questionnaire for Health post (To be collected from HEW)

| **General Information related questions** | | | | | | | | | | | | **Skip** | | **Code** |
| --- | --- | --- | --- | --- | --- | --- | --- | --- | --- | --- | --- | --- | --- | --- |
| 201 | | Name of the kebelle? | ……………. | | | | | | | | |  | |  |
| 202 | | Name of the Health Post? | ……………. | | | | | | | | |  | |  |
| 203 | | Name of the supervising health center? | ……………. | | | | | | | | |  | |  |
| 204 | | Kebele population | Total………..M………F………..  Women 15-49 yrs.……….  Under five---------- | | | | | | | | |  | |  |
| 205 | | Keble household Size (in 2009 E.C) | (Number-----------) | | | | | | | | |  | |  |
| 206 | | Availability of road in the kebelle? | 1= Yes  2= No | | | | | | | | |  | |  |
| 207 | | Availability of network access in the kebelle? | 1= Yes  2= No | | | | | | | | |  | |  |
| 208 | | Number of HEW | (Number------------) | | | | | | | | |  | |  |
| 209 | | Educational level of HEW? |  | | | | | **HEW** | | | |  | |  |
|  |  |  |  |  |  |  |  | 1 | 2 | 3 | 4 |  |  |  |
|  |  |  | Certificate | | | | |  |  |  |  |  |  |  |
|  |  |  | Diploma(HEW) | | | | |  |  |  |  |  |  |  |
|  |  |  | Diploma(Nurse) | | | | |  |  |  |  |  |  |  |
| 210 | | Training status of HEW on MPNDSR? | Trained | | | | |  |  |  |  |  | |  |
|  |  |  | Not Trained | | | | |  |  |  |  |  |  |  |
|  |  |  | I don’t remember | | | | |  |  |  |  |  |  |  |
| 211 | | Work Experience in complete years? | Write in complete in each HEW | | | | |  |  |  |  |  | |  |
| 212 | | Number of WDG in the Kebele(in 2009 E.C) | Total(1:30=_________)  Total(1:5=__________) | | | | | | | | |  | |  |
| **Death Identification and notification related questions** | | | | | | | | | | | | | | |
| 213 | | Was there maternal death in 2009 E.C in your kebelle? | | | 1= Yes  2= No | | | | | | | |  |  |
| 214 | | If yes Q213, How many maternal deaths did you have in your kebelle in 2009 E.C? | | | ………(Number) | | | | | | | |  |  |
| 215 | | If yes Q213, How did you identify the maternal deaths occurred in your kebele?  **(fill for each death from alternative )**  Death 1----------  Death 2---------  Death 3: ---------- | | | 1= By Home to home visit / self  2= By WDG leaders report  3= Steering committee members  4=by family members  5= Other specify…………….. | | | | | | | |  |  |
| 216 | | If yes Q313, after how many hours or days did you identified maternal deaths occurred in your kebele? | | | Death 1: …/……..(Hrs/days)  Death 2: …/……...(Hrs/days)  Death 3: ……/…..(Hrs/days) | | | | | | | |  |  |
| 217 | | Did health post notify maternal death to health facility formally **(Annex 1A)?** | | | 1= Yes  2= No | | | | | | | | If 2→221 |  |
| 218 | | If yes Q217, after how many hours or days of death did you notify formally for the maternal death occurred in your kebelle? **(Annex 1A)?** (See the document) | | | Death 1: ………..(Hours/……..days)  Death 2: ………...(Hours/……days)  Death 3: ………..(Hours/……..days) | | | | | | | |  |  |
| 219 | | If Q217 yes, is there any missed variable in the filled notification format for maternal death? (Observation) | | | 1= Yes  2= No | | | | | | | |  |  |
| 220 | | If Q 219 yes, how many formats are with missed variable in 2009 E.C? | | | ………(Number ) | | | | | | | |  |  |
| 221 | | Was there perinatal death in 2009 E.C in your kebelle? | | | 1= Yes  2= No | | | | | | | | If 2→229 |  |
| 222 | | If Q 221 yes, how many community perinatal deaths did you have in your kebelle in 2009 E.C? | | | Still birth...…….(Number) | | | | | | | |  |  |
|  |  |  |  |  | Early neonatal………(Number) | | | | | | | |  |  |
|  |  |  |  |  | Late neonatal…….……(Number) | | | | | | | |  |  |
| 223 | | If Q 221 yes, how did you identify the community perinatal deaths Occurred in your kebele?**(fill for each death from alternative )**  Death 1----------  Death 2---------  Death 3: ---------- | | | 1= By Home to home visit / self  2= By WDG leaders report  3= Steering committee members  4=by family members  5= Other specify…………….. | | | | | | | |  |  |
| 224 | | If Q 221 yes, after how many hours or days did you identified the community perinatal deaths occurred in your kebele? | | | Death 1: …/……..(Hrs/days)  Death 2: …/……...(Hrs/days)  Death 3: ……/…..(Hrs/days) | | | | | | | |  |  |
| 225 | | Did you notify formally **(Annex 1B)** for community perinatal death occurred in your kebelle? (See the document) | | | 1= Yes  2= No | | | | | | | | If 2→229 |  |
| 226 | | If Q225 yes, after how many hours or days of death did you formally notify for the perinatal death occurred in your kebelle? (See the document) | | | Death 1: …/……..(Hrs/days)  Death 2: …/……...(Hrs/days)  Death 3: ……/…..(Hrs/days) | | | | | | | |  |  |
| 227 | | If Q225 yes, is there any missed variable in the filled notification format for perinatal death? (Observation) | | | 1= Yes  2= No | | | | | | | |  |  |
| 228 | | If Q 227yes, how many formats are with missed variable in 2009 E.C? | | | ………(Number ) | | | | | | | |  |  |
| **Death reviewing related questions** | | | | | | | | | | | | | | |
| 229 | Did you conduct verbal autopsy **(Annex 4A)** for maternal death occurred in your kebelle? | | | | | 1= Yes  2= No | | | | | | | If 2→233 |  |
| 230 | If Q229 yes, after how many weeks of death did conduct verbal autopsy? | | | | | Death 1: ………..(WKS)  Death 2: ………...(Wks.)  Death 3: ………..(Wks.)  Death 4:…………(WKs | | | | | | |  |  |
| 231 | If yes Q229, is there any missed variable in the filled verbal autopsy format for perinatal death? (Observation ) | | | | | 1= Yes  2= No | | | | | | |  |  |
| 232 | If yes Q231, how many formats for community maternal verbal autopsy are with missed variable in 2009 E.C? | | | | | ………(Number ) | | | | | | |  |  |
| 233 | Did you conduct verbal autopsy **(Annex 4B)** for community perinatal death occurred in your kebelle? | | | | | 1= Yes  2= No | | | | | | | If 2→237 |  |
| 234 | If Q233 yes, after how many days of death did conduct verbal autopsy? (See the document) | | | | | Death 1: ………..(days)  Death 2: ………...(days)  Death 3: ………..(days)  Death 4:…………(days) | | | | | | |  |  |
| 235 | If yes Q233, how many formats for community perinatal verbal autopsy are with missed variable in 2009 E.C? | | | | | 1= Yes  2= No | | | | | | |  |  |
| 236 | If Q235 yes Q233, how many formats for community perinatal verbal autopsy are with missed variable in 2009 E.C? | | | | | ………(Number ) | | | | | | |  |  |
| 237 | Did HEW participate in death review process conducted in your supervising health center? | | | | | 1= Yes  2= No | | | | | | | If 2→239 |  |
| 238 | If Q237 yes, in how many death review process did you participate in 2009 EC? | | | | | -----------(Number) | | | | | | |  |  |
| 239 | Did the Kebelle’s administrator participate in the death review process? | | | | | 1= Yes  2= No | | | | | | | If 2→241 |  |
| 240 | If Q239 yes, in how many of them did he/she participate in 2009 EC? | | | | | maternal death …….(Number)  Perinatal death ………(Number)  I don’t remember……… | | | | | | |  |  |
| **Report and Surveillance related questions** | | | | | | | | | | | | | | |
| 241 | | How many weekly surveillance reports did you have in in 2009 EC? (See the document) | | | | ………….(Number) | | | | | | | If 2→244 |  |
| 242 | | Did the health post have timely surveillance report (Monday to Sunday) (See the document) | | | | 1= Yes  2= No | | | | | | |  |  |
| 243 | | If Q242 yes, how many weekly reports did you report timely in 2009 EC?  **(See the document)** | | | | …………(Number) | | | | | | |  |  |
| 244 | | Did you send weekly zero report regarding maternal death to health center? | | | | 1= Yes  2= No | | | | | | | If 2→246 |  |
| 245 | | If yes, Number of zero weekly reporting 2009 EC? | | | | ………….(Number) | | | | | | |  |  |
| 246 | | Did you send weekly zero report regarding perinatal death to health center? | | | | 1= Yes  2= No | | | | | | | If 2→248 |  |
| 247 | | If Q246 yes, Number of zero weekly reports in 2009 EC? | | | | ………….(Number) | | | | | | |  |  |
| 248 | | Did the Kebelle’s WDG send weekly report including zero report regarding maternal and prenatal death to health post? | | | | 1= Yes  2= No | | | | | | | If 2→251 |  |
| 249 | | If yes Q248, How? | | | | ………………………………  ………………………………..  ……………………………….. | | | | | | |  |  |
| 250 | | If Q No 248 is yes, the numbers of WDG send weekly reporting 2009 EC? (See the document) | | | | ………….(Number) | | | | | | |  |  |
| 251 | | Availability of uninterrupted registering of pregnant mothers?(See the document) | | | | 1= Yes  2= No | | | | | | |  |  |
| **Death response related questions** | | | | | | | | | | | | | | |
| 252 | | Does the health post have an action plan which is developed after maternal death review? (See Documents) | | | | | 1= Yes  2= No | | | | | | If 2→254 |  |
| 253 | | If Q252 yes, how many action plans developed after maternal death review in 2009 EC? (See the document) | | | | | ………….(Number) | | | | | |  |  |
| 254 | | Does the health post have an action plan which is developed after perinatal death review? | | | | | 1= Yes  2= No | | | | | | If 2→256 |  |
| 255 | | If Q254 yes, how many action plans developed after perinatal death review in 2009 EC? (See the document) | | | | | ………….(Number) | | | | | |  |  |
| 256 | | Did the Kebelle’s steering committee participate in the death response discussion? | | | | | 1= Yes  2= No | | | | | | If 2→259 |  |
| 257 | | If Q256 yes, what issues was discussed? | | | | | ……………………..  ……………………  …………………… | | | | | |  |  |
| 258 | | If Q No 256 is yes, in how many of the death response process did the committee participate in 2009 EC? | | | | | ………...(Number) | | | | | |  |  |
| 259 | | Did you discussed about the main cause of the death with WDA members so as to prevent further death? (see document) | | | | | 1= Yes  2= No | | | | | | If 2→261 |  |
| 260 | | If Q259 yes, what issues were raised? (Multiple responses are possible) | | | | | 1= Discussion on the importance of ANC service  2= Discussion on the importance of skilled delivery  3= Discussion on the importance of PNC service  4= Discussion on the importance of birth preparedness and complication readiness  5= Discussion on the importance of family planning service  6= Discussion on pregnancy related danger signs  7= Discussion on perinatal period danger signs  8= Others (……………………...) | | | | | |  |  |
| **Availability of registrations and formats related questions** | | | | | | | | | | | | | | |
| 261 | | Availability of rumor logbook?  (By observation ) | | 1= Yes  2= No | | | | | | | | |  |  |
| 262 | | Check the availability of formats /annexes and circle all available formats | | 1.Maternal Death Notification format  2.Perinatal Death Notification format  3.Maternal verbal Autopsy format  4. prenatal verbal Autopsy format | | | | | | | | |  |  |
| 263 | | Availability of maternal death registration format? (By observation ) | | 1= Yes  2= No | | | | | | | | |  |  |
| 264 | | Availability of perinatal death registration format? (By observation ) | | 1= Yes  2= No | | | | | | | | |  |  |
| 265 | | Availability of weekly surveillance report format? (By observation ) | | 1= Yes  2= No | | | | | | | | |  |  |

**I thank you!**

**ናይ ጥዕና ኬላ መሕትት**

**ቅጥዒ ሓበሬታመውሃቢን ዉዕል ስምምዕነትን**

**መእተዊ፡-**

ጥዕና ይሃበለይ! ሽመይ…………….ይበሃል:: ኢንስቲትዩት ምርምር ጥዕና ትግራይ ምስ ቢሮ ሓለዋ ጥዕና ክልል ትግራይን UNFPAን ብምትሕግጋዝ ንትግበራ ፈተሸ ሞት ኣዴታትን ሕንጦታትን ምላሽ ምሃብን ፕሮግራም ንዘካይድዎ ናይ መፅናዕቲ ሓበሬታ ንምእካብ እየ መፂአ፡፡ እዚ መፅናዕቲ ብትግራይ ደረጃ ኣብ ዝተመረፃ 22 ወረዳታት ኣብ ዝርከባ ጥዕና ትካላት ዝካየድ እንትኸውን ትካልኩም/ክን ድማ ካብተን ዝተመረፃ ትካላት ሓንቲ እያ፡፡ ኣብዚ መፅናዕቲ ዝሳተፉ ድማ ሓለፍቲ ትካላት፤ ናይ ሰርቨይላንስ ኪኢላታትን ጥሙር ጥዕና ሰራሕተኛታትን እዮም። ተሳትፎኹም/ክን ነዚ ኣብ ፈተሸ ሞት ኣዴታትን ሕንጦታትን ምላሽ ምሃብን አመልኪቱ ንዝካየድ መፅናዕቲ ብጣዕሚ አገዳሲ እዩ፡፡ እዚ ቃለ መሕተት ንኣስታት ሓደ ሰዓት ዝወስድ እንትኸውን ሽሞም/ምክን ኣይምዝገብን፡፡ እትህቡና/ባና ሓበሬታ ምስጢራዊ እዩ፡፡ብምስታፍኩም/ኽን ዘምፅአልኩም/ክን ሳዕቤን የብሉን፡፡ምስታፍኩም/ክን ብድልየት ዝተመስረተ እትኸውን ክትምልስዎ/ኦ ዘይትድልይዎ/ኦ ሕቶ ምዝላል ትኽእሉ/ላ ኢኹም/ክን፡፡

ኣብዚ መፅናቲ ንምስታፍ ፍቓደኛ ዲኹም/ኽን?

- ኣይኮንኩን…………….(“የቐንየለይ፡፡” ብምባል እዚ ቓለ-መሕተት የቛርፁ/ፃ::)
- እወ………………. (“የቐንየለይ::” ብምባል ንተሓታቲ ድሕሪ ምፍራም ቃለ መሕተቶም/ን ይቐፅሉ/ላ፡፡)

ፌርማ ተሓታቲ፡--------------------------------

ሓላፍነት ተሓታቲ፡-------------------------------

ሽም ሓታቲ ----------------------------- ፌርማ----------------ዕለት----------------

መፍለጢ ቑፅሪ መሕትት------------------

ነዚ ዘረጋገፀ ተቆፃፃሪ ሽም ------------------------ፌርማ---------------- ዕለት----------------

**ኢንስቲትዩት ምርምር ጥዕና ትግራይ**

**ብርኪ ትግበራ ፈተሸ ሞት ኣዴታትን ሕንጦታትን ምላሽ ምሃብን ኣብ ትግራይ፣ ሰሜን ኢትዮጵያ።**

**ክፍሊ I: ናይ ጥዕና ኬላ መሕትት (ካብ ጥሙር ጥዕና ሰራሕተኛ ዝእከብ ሓበሬታ)**

| **ሓፈሻዊ ሓበሬታ መሕትት ንጥሙር ጥዕና ሰራሕተኛ** | | | | | | | | | | **ዝለል** | | **ኮድ** |
| --- | --- | --- | --- | --- | --- | --- | --- | --- | --- | --- | --- | --- |
| 201 | ሽም ጣብያ? | | ……………. | | | | | | |  | |  |
| 202 | ሽም ኬላ ጥዕና? | | ……………. | | | | | | |  | |  |
| 203 | ሽም ክላስተር ጣብያ ጥዕና? | | ……………. | | | | | | |  | |  |
| 204 | በዝሒ ህዝቢ እቲ ጣብያ?  ኣብ (2009 ዓ/ም) | | ጠቅላላ በዝሒ ህዝቢ -------  ተባ…… ኣን……………..  ደቂ ኣንስትዮ 15-49 ዓመት…….  ትሕቲ 5 ዓመት ---------- | | | | | | |  | |  |
| 205 | በዝሒ መራሕቲ ስድራ? | | (ብቁፅሪ -----------)2009 ዓ/ም) | | | | | | |  | |  |
| 206 | ኣብዚ ጣብያ ዘእቱ ፅርጊያ ኣሎ ዶ? | | 1= እወ  2= ኣይፋል | | | | | | |  | |  |
| 207 | ስልኪ ኔት ወርክ ኣብቲ ጣብያ ይሰርሕ ዶ? | | 1= እወ  2= ኣይፋል | | | | | | |  | |  |
| 208 | በዝሒ ጥሙር ጥዕና ሰራሕተኛታት | | (ብቁፅሪ -----------) | | | | | | |  | |  |
| 209 | ብርኪ ትምህርቲ ሰራሕተኛታት ጥሙር ጥዕና? (ኣብ ሕድሕድ HEW X ምልክት ግበር) | |  | | | HEW | | | |  | |  |
|  |  |  |  |  |  | 1 | 2 | 3 | 4 |  |  |  |
|  |  |  | ግዝያዊ | | |  |  |  |  |  |  |  |
|  |  |  | ሰርተፊኬት | | |  |  |  |  |  |  |  |
|  |  |  | ዲፕሎማ | | |  |  |  |  |  |  |  |
|  |  |  | ዲፕሎማ/ነርስ | | |  |  |  |  |  |  |  |
| 210 | ኩነታት ስልጠና ሰራሕተኛታት ጥሙር ጥዕና ኣብ MPNDSR? | | ዝሰልጠነት | | |  |  |  |  |  | |  |
|  |  |  | ዘይሰልጠነት | | |  |  |  |  |  |  |  |
|  |  |  | ኣይዝክርን | | |  |  |  |  |  |  |  |
| 211 | ስራሕ ልምዲ **HEW** ብሙሉእ ዓመት?  **(ኣብ ሕድሕድ HEW ብሙሉእ ዓመት ፀሓፍ)** | | | | |  |  |  |  |  | |  |
| 212 | ናይ ጣብያ በዝሒ ል/ጉጅለ? | | ጠቕላላ (1:30=____) (1:5=_____) | | | | | | |  | |  |
| **ምልላይ ምፍላጥን ሞት ዝምልከት መሕትት** | | | | | | | | | | | | |
| 213 | ኣብ ጣብያክን ኣብ 2009 ዓ.ም ሞት ኣዶ ኣጋጢሙ ኔሩ ዶ? | | | | 1= እወ  2= ኣይፋል | | | | | | መልሲ2→221 |  |
| 214 | ንሕቶ ቁ 213 መልስክን እወ እንተኾይኑ ኣብ 2009 ዓ.ም ኣብ ጣብያኩም ክንደይ ሞት ኣዴታት ኣጋጢሙ? | | | | ……… (ብቑፅሪ) | | | | | |  |  |
| 215 | ኣብ ጣብያኽን ዘጋጠመ ሞት ኣዴታት ንመጀመርያ ግዜ ብኸመይ ፈሊጥክንአን?  **(ካብ ዘለዉ መማረፅታት ቑፅሪ ኣብ ሕድሕድ ሞት ይመላእ)**  ሞት 1----------  ሞት 2----------  ሞት 3---------- | | | | 1= ገዛ ንገዛ አእንዘረሉ/ ባዕልና  2=ብልምዓት ጉጅለ መራሕቲ ሪፖርት  3= ካብ ስትሪንግ ኮሚቴ ኣባለት  4= ካብ ኣባላት ስድራ መዋቲት  5= ብኻሊእ (ይገለፅ) ……….. | | | | | |  |  |
| 216 | ኣብ ጣብያኽን ንዘጋጠመ ሞት ኣዴታት ድሕሪ ክንደይ ሰዓታት ወይ መዓልትታት ፈሊጥክንአን? | | | | ሞት1: …../….(ሰዓታት/መዓልቲ)  ሞት2: …../….(ሰዓታት/መዓልቲ)  ሞት3 :…/….(ሰዓታት/መዓልቲ) | | | | | |  |  |
| 217 | ኣብ ጣብያኽን ዘጋጠመ ናይ ኣዴታት ሞት ንልዕለይኽን ዘሎ ጥዕና ትካል ብስሩዕ ቕጥዒ ኣፍሊጥክን ዶ? **(Annex1A፡ ደኩሜንት ይረአ)** | | | | 1= እወ  2= ኣይፋል | | | | | | መልሲ2  221 |  |
| 218 | ንሕቶ ቁ 217 መልስኽን እወ እንተኾይኑ ድሕሪ ክንደይ ሰዓታት ወይ መዓልታት ሙማተን ንልዕለይኽን ዘሎ ጥዕና ትካል ብስሩዕ ቕጥዒ ኣፍሊጥክን? **(Annex1A፡ደኩሜንት ይረአ)** | | | | ሞት1: …../….(ሰዓታት/መዓልቲ)  ሞት2: …../….(ሰዓታት/መዓልቲ)  ሞት3 :…/….(ሰዓታት/መዓልቲ) | | | | | |  |  |
| 219 | ቁ 217 መልሱ እወ እንተኾይኑ ካብቶም ዝተለኣኹ መፍለጢ ቕጥዕታት ምሉእነት ዘይብሎም ቕጥዕታት ኣለዉ ዶ? **(ምጉዳል ሙሉእነት ቕጥዕታት ረአ)** | | | | 1= እወ  2= ኣይፋል | | | | | |  |  |
| 220 | ንሕቶ ቁ 219 መልስኽን እወ እንተኾይኑ ኣብ 2009 ዓ/ም ካብ ዝተለኣኹ ክንደይ ምሉእነት ዘይብሎም ቕጥዒ መፍለጢ ሞት ኣዴታት ኣለዉ? | | | | ……(ቁፅሪ) | | | | | |  |  |
| 221 | ኣብ ጣብያኽን ኣብ 2009 ዓም ሞይቶም ዝተወለዱ ወይ ምስ ተወለዱ ዝሞቱ ሕንጦታት ኣብ ሕ/ሰብ ኣጋጢሙ ኔሩ ዶ? | | | | 1= እወ  2= ኣይፋል | | | | | | መልሲ2→229 |  |
| 222 | ንሕቶ ቁ 221 መልስኽን እወ እንተኾይኑ ክንደይ ሞይቶም ዝተወለዱን ምስ ተወለዱ ዝሞቱን ሕንጦታት ኣብ ሕ/ሰብ ኣጋጢሙ? **(ደኩሜንት ይረአ)** | | | | ሞይቶም ዝተወለዱ……(ብቁፅሪ) | | | | | |  |  |
|  |  |  |  |  | ካብ ዝውለዱ ኣብ ዉሽጢ ሐደ ሰሙን ዝሞቱ…………(ብቁፅሪ) | | | | | |  |  |
|  |  |  |  |  | ካብ ዝውለዱ ኣብ ዉሽጢ 7-28 መዓልቲ ዝሞቱ……… (ብቁፅሪ) | | | | | |  |  |
| 223 | ኣብ ጣብያኽን ኣብ ሕ/ሰብ ዘጋጠመ ሞት ሕንጦታት ንመጀመርያ ግዜ ብኸመይ ፈሊጥክንኦም ?**(ካብ ዘለዉ መማረፅታት ቑፅሪ ኣብ ሕድሕድ ሞት ይመላእ)**  ሞት 1----------  ሞት 2----------  ሞት 3----------  ሞት 4----------  ሞት 5----------  ሞት 6----------  ሞት 7---------- | | | | 1= ገዛ ንገዛ ብምዝዋር /ባዕልና  2= ብልምዓት ጉጅለ መራሕቲ ሪፖርት  3= ካብ ስትሪንግ ኮሚቴ ኣባላት  4= ካብ ኣባላት ስድራ  5= ኻሊእ (ይገለፅ) ……….. | | | | | |  |  |
| 224 | ኣብ ጣብያኽን ዘጋጠመ ኣብ ሕ/ሰብ ሞይቶም ዝተወለዱን ምስ ተወለዱ ዝሞቱን ሕንጦታት ድሕሪ ክንደይ ሰዓታት ወይ መዓልትታት ፈሊጥክንኦም? | | | | ሞት1: …../….(ሰዓታት/መዓልቲ)  ሞት2: …../….(ሰዓታት/መዓልቲ)  ሞት3 :…/….( ሰዓታት/መዓልቲ) | | | | | |  |  |
| 225 | ኣብ ጣብያኽን ዘጋጠመ ኣብ ሕ/ሰብ ሞይቶም ዝተወለዱን ምስ ተወለዱ ዝሞቱን ሕንጦታት ንልዕለይኽን ዘሎ ጥዕና ትካል ብስሩዕ ቕጥዒ ኣፍሊጥክን ዶ? **(ደኩሜንት ይረአ)** | | | | 1= እወ  2= ኣይፋል | | | | | | መልሲ2→229 |  |
| 226 | ንሕቶ ቁ 225 መልስኽን እወ እንተኾይኑ ድሕሪ ክንደይ ሰዓታት ወይ መዓልትታት ኣፍሊጥክን? **(ደኩሜንት ይረአ)** | | | | ሞት 1: ……/…… (ሰዓት/መዓልቲ)  ሞት 2: ……/…… (ሰዓት/መዓልቲ)  ሞት 3… /…… (ሰዓት/መዓልቲ) | | | | | |  |  |
| 227 | ንሕቶ ቁ 225 መልስኽን እወ እንተኾይኑ ካብቶም ዝተለኣኹ መፍለጢ ቕጥዕታት ሙሉእነት ዝጎደሎም ቕጥዕታት ኣለዉ ዶ? **(ምጉዳል ሙሉእነት ቕጥዕታት ረአ)** | | | | 1= እወ  2= ኣይፋል | | | | | |  |  |
| 228 | ንሕቶ ቁ 227 መልስኽን እወ እንተኾይኑ ኣብ 2009 ዓ/ም ክንደይ ጉድለት ምሉእነት ዘለዎ ቕጥዒ መፍለጢ ሞት ሕንጦታት ኣሎ? | | | | ……(ቁፅሪ) | | | | | |  |  |
| **ግምገማ ሞት ዝምልከት መሕትት(Death Reviewing)** | | | | | | | | | | | | |
| 229 | ኣብ ጣብያኽን ንዘጋጠመ ሞት ኣዴታት ሓበሬታ ምክንያት ሞት (verbal autopsy) ትወስዳ ዶ? | | | | 1= እወ  2= ኣይፋል | | | | | | መልሲ2→233 |  |
| 230 | ንሕቶ ቁ 229 መልሲ እወ እንተኾይኑ ኣብ ጣብያኽን ንዘጋጠመ ሞት ኣዶ ድሕሪ ክንደይ ሰሙናት ሞት ኣዶ ሓበሬታ ምክንያት ሞት (verbal autopsy) ወሲድክን?  **(Annex 4A: ደኩሜንት ይረአ)** | | | | ሞት 1: ……….. (ሰሙናት)  ሞት 2: ………….. (ሰሙናት)  ሞት 3: ………….. (ሰሙናት)  ሞት 4: ………….. (ሰሙናት) | | | | | |  |  |
| 231 | ንሕቶ ቁ 229 መልስኽን እወ እንተኾይኑ ካብቶም ዝተለኣኹ ሞት ኣዶ (verbal autopsy) ቕጥዕታት ሙሉእነት ዝጎደሎም ቕጥዕታት ኣለዉ ዶ? **(Annex 4A: ምጉዳል ሙሉእነት ቕጥዕታት ረአ)** | | | | 1= እወ  2= ኣይፋል | | | | | |  |  |
| 232 | ንሕቶ ቁ 231 መልስክን እወ እንተኾይኑ ኣብ 2009 ዓ/ም ክንደይ ጉድለት ምሉእነት ዘለዎ ቕጥዒ verbal autopsyኣሎ? | | | | ……(ቁፅሪ) | | | | | |  |  |
| 233 | ኣብ ጣብያኽን ኣብ ሕ/ሰብ ሞይቶም ዝተወለዱን ምስ ተወለዱ ዝሞቱን ሕንጦታት ሓበሬታ ምክንያት ሞት (verbal autopsy) ትወስዳ ዶ? | | | | 1= እወ  2= ኣይፋል | | | | | | መልሲ2→237 |  |
| 234 | ንሕቶ ቁ 233 መልሲ እወ እንተኾይኑ ኣብ ሕ/ሰብ ሞይቶም ዝተወለዱን ምስ ተወለዱ ዝሞቱን ሕንጦታት ድሕሪ ክንደይ መዓልታት verbal autopsy ሓበሬታ ወሲድክን? **(Annex 4B: ደኩሜንት ይረአ)** | | | | ሞት 1: ……….. (መዓልታት)  ሞት 2: ……….. (መዓልታት)  ሞት 3: ……….. (መዓልታት)  ሞት 4: ……….. (መዓልታት) | | | | | |  |  |
| 235 | ንሕቶ ቁ 233 መልስኩም/ክን እወ እንተኾይኑ ካብቶም ዝተለኣኹ verbal autopsy ቕጥዕታት ሙሉእነት ዝጎደሎም ቕጥዕታት ኣለዉ ዶ? **(Annex 4B: ምጉዳል ሙሉእነት ቕጥዕታት ረአ)** | | | | 1= እወ  2= ኣይፋል | | | | | |  |  |
| 236 | ንሕቶ ቁ 235 መልስኽን እወ እንተኾይኑ ኣብ 2009 ዓ/ም ክንደይ ሙሉእነት ዝጎደሎም ቕጥዒ verbal autopsy ሞት ሕንጦታት ኣሎ? | | | | ……(ቁፅሪ) | | | | | |  |  |
| 237 | ኣብቲ ዝካየድ ናይ ጣብያ ጥዕናኽን ከይዲ ገምጋም ምክንያት ሞት ኣዴታትን ሕንጦታትን ትሳተፋ ዶ? **(ካበተን ዘለዋ ጥሙር ጥዕና ሰራሕተኛታት ዋላ ሓንቲ)** | | | | 1= እወ  2= ኣይፋል | | | | | | መልሲ2→239 |  |
| 238 | ንሕቶ ቁ 237 መልሲ እወ እንተኾይኑ ኣብ 2009 ዓ.ም ኣብ ክንደይ ገምጋም ምክንያት ሞት ተሳቲፍክን? | | | | ኣብ ኣዴታት………..(ብቑፅሪ)  ኣብ ሕንጦታት………..(ብቑፅሪ) | | | | | |  |  |
| 239 | ኣቦ ወንበር ጣብያ ኣብቲ ገምጋም ምኽንያት ሞት ይሳተፍ ዶ? | | | | 1= እወ  2= ኣይፋል | | | | | | መልሲ2→241 |  |
| 240 | መልሲ ቁ 239 እወ እንተኾይኑ ኣብ 2009 ዓ.ም አብ ክንደይ ገምጋም ምክንያት ሞት ተኻፊሉ/ላ? | | | | ኣብ ኣዴታት………..(ብቑፅሪ)  ኣብ ሕንጦታት………..(ብቑፅሪ)  ኣይዝክርን……………. | | | | | |  |  |
| **ሪፖርትን ሰርቪላንስን ዝምልከት መሕትት** | | | | | | | | | | | | |
| 241 | ኣብ 2009 ዓ.ም በዝሒ ዝተለኣኹ ሰሙናዊ ናይ ሰርቪላንስ (surveillance) ሪፖርት?  **ደኩሜንት ይረአ)** | | | ……… (ብቑፅሪ) | | | | | | |  |  |
| 242 | ካብ ጥዕና ኬላኽን እዋናዊ ሰሙናዊ ሪፖርት ትልእኻ ዶ? (ካብ ሶኒ ክሳብ ሰንበት) **(ደኩሜንት ይረአ)** | | | 1= እወ  2= ኣይፋል | | | | | | | መልሲ2→244 |  |
| 243 | ንሕቶ ቁ 242 መልሲ እወ እንተኾይኑ ኣብ 2009 ዓ.ም ክንደይ ሰሙናዊ ሪፖርት ብእዋኑ ተላኢኹ? **(ደኩሜንት ይረአ)** | | | ……… (ብቑፅሪ) | | | | | | |  |  |
| 244 | ብዛዕባ ሞት ኣዴታት ሰሙናዊ ዜሮ ሪፖርት ናብ ልዕለይኽን ዘሎ ጥዕና ትካል ትሰዳ ዶ? | | | 1= እወ  2= ኣይፋል | | | | | | | መልሲ2→246 |  |
| 245 | ንሕቶ ቁ 244 መልስኽን እወ እንተኾይኑ ኣብ 2009 ዓ.ም ክንደይ ዜሮ ሰሙናዊ ሪፖርት ሞት ኣዶ ሰዲድክን? **(ደኩሜንት ይረአ)** | | | ……… (ብቑፅሪ) | | | | | | |  |  |
| 246 | ብዛዕባ ሞይቶም ዝተወለዱ ወይ ምስ ተወለዱ ዝሞቱ ሕንጦታት ሰሙናዊ ዜሮ ሪፖርት ናብ ልዕለይኽን ዘሎ ጥዕና ትካል ትሰዳ ዶ? | | | 1= እወ  2= ኣይፋል | | | | | | | መልሲ2→248 |  |
| 247 | ንሕቶ ቁ 246 መልስኽን እወ እንተኾይኑ ኣብ 2009 ዓ.ም ክንደይ ዜሮ ሰሙናዊ ሪፖርት ሞት ሕንጦታት ሰዲድክን? **(ደኩሜንት ይረአ)** | | | ……… (ብቑፅሪ) | | | | | | |  |  |
| 248 | ኣብ ጣብያኽን ዘለዋ ልምዓት ጉጅለ ብዛዕባ ሞት ኣዴታትን ሕንጦታትን ሰሙናዊ ሪፖርት (ዜሮ ሪፖርት ዝሓወሰ) ናብ ኬላ ጥዕና ይሰዳ ዶ? | | | 1= እወ  2= ኣይፋል | | | | | | | መልሲ2→251 |  |
| 249 | ንሕቶ ቁ 248 መልስኽን እወ እንተኾይኑ ብኸመይ ይሰዳ? | | | ………………………………  ……………………………….. | | | | | | |  |  |
| 250 | ንሕቶ ቑፅሪ 248 መልስኽን እወ እንተኾይኑ ኣብ 2009 ዓ.ም በዝሒ ልሞዓት ጉጅለ ሰሙናዊ ሪፖርት ዝሰደዳ?  **(ደኩሜንት ረአ)** | | | ዜሮ ሪፖርት ሞት ኣዶ ዝሰዳ ል/ጉጅለ …… (ብቑፅሪ)  ዜሮ ሪፖርት ሞት ሕንጦ  ዝሰዳ ል/ጉጅለ …… (ብቑፅሪ) | | | | | | |  |  |
| 251 | ኣብ ጣብያኽን ዘይተቖራረፀ ምዝገባ ጥኑሳት ተካይዳ ዶ?**(ደኩሜንት ይረአ)** | | | 1= እወ  2= ኣይፋል | | | | | | |  |  |
| **ምላሽ ምሃብ ዝምልከት መሕትት** | | | | | | | | | | | | |
| 252 | ድሕሪ ገምጋም ሞት ኣዴታት ዝተዳለወ መተግበሪ ትልሚ ኣሎ ዶ? **(Annex 7:ደኩሜንት ይረአ)** | | | 1= እወ  2= ኣይፋል | | | | | | | መልሲ2→254 |  |
| 253 | ንሕቶ ቁ 252 መልስኽን እወ እንተኾይኑ ኣብ 2009 ዓ.ም ድሕሪ ገምጋም ሞት ኣዴታት ክንደይ መተግበሪ ትልሚ ተዳልዩ? **(ደኩሜንት ይረአ)** | | | ……… (ብቑፅሪ) | | | | | | |  |  |
| 254 | ድሕሪ ገምጋም ሞይቶም ዝተወለዱን ምስ ተወለዱ ዝሞቱን ሕንጦታት ዝተዳለወ መተግበሪ ትልሚ (action plan) ኣሎ ዶ? **(Annex 7:ደኩሜንት ይረአ)** | | | 1= እወ  2= ኣይፋል | | | | | | | መልሲ2→256 |  |
| 255 | ንሕቶ ቁ 254 መልስኽን እወ እንተኾይኑ ኣብ 2009 ዓ.ም ድሕሪ ገምጋም ክንደይ መተግበሪ ትልሚ (action plan) ተዳልዩ? **(Annex 7:ደኩሜንት ይረአ)** | | | ……… (ብቑፅሪ) | | | | | | |  |  |
| 256 | ናይ ጣብያኹም ስትሪንግ ኮሚቴ ካሊእ ሞት ንከየጋጥም ምላሽ ኣብ ምሃብ (response) ይመያየጡ ዶ? | | | 1= እወ  2= ኣይፋል | | | | | | | መልሲ2→259 |  |
| 257 | ንሕቶ ቁ 256 መልስኽን እወ እንተኾይኑ እንታይ መፍትሒ ሓሳባት ኣቕሪቦም?  **(ቃለ ጉባኤ ይረአ)** | | | …………………………………  …………………………………………………………………… | | | | | | |  |  |
| 258 | ንሕቶ ቁፅሪ 256 መልስኽን እወ እንተኾይኑ ኣባላት ስትሪንግ ኮሚቴ ኣብ 2009 ዓ.ም ኣብ ክንደይ ምይይጥ ምላሽ ምሃብ (response) ተሳቲፎም? | | | ……… (ብቑፅሪ) | | | | | | |  |  |
| 259 | ምስ ልሞዓት ጉጅለ ካሊእ ሞት ንኸየጋጥም አብ ዋና ዋና ምክንያታት ሞት ተመያይጥክናሎም ዶ?  **(ቃለ ጉባኤ ይረአ)** | | | 1= እወ  2= ኣይፋል | | | | | | | መልሲ2→261 |  |
| 260 | ንሕቶ ቁፅሪ 259 መልስኽን እወ እንተኾይኑ እንታይ ሓሳባት ተመያይጥክን?  **(ቃለ ጉባኤ ይረአ)** | | | 1=ኣብ ኣድላይነት ግልጋሎት ቅድመ ወሊድ( ANC )  2= ኣብ ኣድላይነት ግልጋሎት ወሊድ ብበዓል ሞያ  3=ኣብ ኣድላይነት ግልጋሎት ድሕረ ወሊድ(PNC)  4= ኣብ ኣድላይነት ግልጋሎት ምድላው ወሊድን ኣብ ወሊድ ከጋጥሙ ዝክእሉ ሓደጋታትን  5=ኣብ ኣድላይነት ግልጋሎት ምጣነ ስድራ  6=ኣብ ኣድላይነት ግልጋሎት ምስ ጥንሲ ዝተተሓሓዙ ምልክታት ሓደጋ  7=ኣብ ኣድላይነት ግልጋሎት ምልክታት ሓደጋ እዋን ቅድመ ወሊድ  8= ካልኦት (………………...) | | | | | | |  |  |
| **ህላወ መዛግብትን ናይ ሪፖርት ቅጥዕታትን ዝምልከት መሕትት( ብምርኣይ ጥራሕ ዝምለሱ)** | | | | | | | | | | | | |
| 261 | ናይ ጭምጭምታ መዝገብ ኣሎ ዶ? | 1= እወ  2= ኣይፋል | | | | | | | | |  |  |
| 262 | እዞም ዝስዕቡ ልጋባት/ቕጥዕታት ምህላዎም ብምርግጋፅ ዘለዉ ኩሎም ይከበበሎም | 1 = ሞት ኣዶ መፍለጢ ቅጥዒ(Annex_1A)  2= ሞት ሕንጦ መፍለጢ ቅጥዒ(Annex_1B)  3= ምክንያት ሞት ኣዶ ድሕሪ ሞት መመርመሪ ቅጥዒ/Annex _4A: Maternal verbal Autopsy format  4= ምክንያት ሞት ሕንጦ ድሕሪ ሞት መመርመሪ ቅጥዒ/Annex_4B: prenatal verbal Autopsy format | | | | | | | | |  |  |
| 263 | መመዝገቢ ቅጥዒ ሞት ኣዴታት ኣሎ ዶ? | 1= እወ  2= ኣይፋል | | | | | | | | |  |  |
| 264 | መመዝገቢ ቅጥዒ ሞት ሕንጦ ኣሎ ዶ? | 1= እወ  2= ኣይፋል | | | | | | | | |  |  |
| 265 | ናይ ሰሙናዊ ሪፖርት ቕጥዒ ኣሎ ዶ ? | 1= እወ  2= ኣይፋል | | | | | | | | |  |  |

**ብጣዕሚ የቐንየለይ!!**
