# Supplementary material for: Maternal and perinatal death surveillance and response in Ethiopia: Achievements, challenges and prospects
Source: PLoS One. 2019 Oct 11;14(10):e0223540. doi: 10.1371/journal.pone.0223540 (PMC6788713; doi:10.1371/journal.pone.0223540)
Supplement: S2 File — (DOCX) [file pone.0223540.s003.docx]

**Health Facility Questionnaire**

**Inform sheet and consent form**

**Introduction**

My name is --------------------------------- I am a member a research team conducting by Tigray Health Research Institute in collaboration with Tigray Regional Health Bureau and UNFPA on **Implementation Status of Maternal, Perinatal and Neonatal Death Surveillance and Response in Tigray Region, Northern Ethiopia**. This study is commencing in health facilities of selected 22 districts of Tigray and your health facility/organization is among these facilities. We are interviewing head of health facilities, HEWs and PHEM focal persons to obtain the information. Your involvement in this research is important to know the implementation status of MPNDSR programme in the region. The questionnaire will take around hour and your name will not be written in this form and will never be used in connection with any of the information you tell me. You do not have to answer any question that you do not want to and you may end this interview at any time you want to. There is no risk for being not participating in the study. However, your participation will help us to know the implementation status of MPNDSR programme. We appreciate your help in responding to this Research questions.

Do have willingness to participate?

No----------- (Say “Thank you!!” and stop here!)

Yes---------- (Say “Thank you!!” and make sure that you got the signature of the participant)

Respondent’s signature-------------------------------------------------------- date----------------

Responsibility of the participant-----------------------------

Name of interviewer ----------------------------- signature-------------------- date----------------

Questionnaire code ------------------

Name of supervisor ------------------------ signature---------------- date----------------

**Tigray Health Research Institute**

**Implementation Status of Maternal, Perinatal and Neonatal Death Surveillance and Response in Tigray Region, Northern Ethiopia**

**Annex II: Questionnaire for health facility level (Part one: to be collected from head of facility)**

| **General information related question** | | | | | Skip | **Code** |
| --- | --- | --- | --- | --- | --- | --- |
| 301 | Name of the district | ………………………….. | | |  |  |
| 302 | Name of the health facility | ………………………… | | |  |  |
| 303 | Type of the health facility? | 1= Health center  2= Primary Hospital  3=General Hospital  4= Referral Hospital | | |  |  |
| 304 | Ownership of the health facility? | 1= Public  2= Private  3= Other (Specify…………. | | |  |  |
| 305 | Cluster population | Total----------M……….F………..  Women 15-49 yrs.……….  Under five---------- | | |  |  |
| 306 | No of HEW in the cluster? (**For Primary hospital and HC only**) | ………….(Number) | | |  |  |
| 307 | No of WDA in the cluster?(**For Primary hospital and HC only** | ………….(Number) | | |  |  |
| 308 | Did the facility Establish MPNDSR (RRT) committee? | 1=Yes  2= No | | | If 2→310 |  |
| 309 | If yes, could you tell me the composition of MPNDSR committee members  (multiple response possible) | 1=Administrative staffs  2=Technical staff  3=Community representation  4= Others ---------- | | |  |  |
| 310 | Number of health professionals trained on MPNDSR in the Facility? (See the document) | Midwife---------(Number)  Nurses----------(Number)  Health officer------(Number)  Emergency surgeon-----(Number)  General practitioner-----(Number)  Gynecologist-------(Number)  Other----------------(Number) | | |  |  |
| 311 | Did the head of health facility trained for MPNDSR? | 1= Yes  2= No | | |  |  |
| 312 | Work experience of the head of health facility in complete years? | (Year----------) | | |  |  |
| 313 | Profession of the MPNDSR (RRT) committee chairman? | ------------------ | | |  |  |
| 314 | Position of MPNDSR (RRT) committee chairman in the health facility? | ------------------- | | |  |  |
| 315 | For how long did the MPNDSR chairman lead the committee?(in complete years) | (Year----------) | | |  |  |
| 316 | Frequency of MPNDSR committee meeting in 2009 E.C? (See the Minute) | ………(Number) | | |  |  |
| 317 | Frequency of cluster review meeting with MPNDSR agenda ?(See the Minute) | ………(Number) | | |  |  |
| 318 | Did you have public conferences in your catchment area raised MPNDSR agenda? | 1= Yes  2= No | | | If 2→320 |  |
| 319 | If yes Q318, how many public conferences conducted in 2009 E.C? | ………(Number) | | |  |  |
| **Reviewing related questions** | | | | | | |
| 320 | Was there maternal death in 2009 E.C in your cluster? | 1= Yes  2= No | | |  |  |
| 321 | If yes Q320, numbers of death in 2009 E.C? (See the document) | At home…………(Number) | | |  |  |
|  |  | At transit ……(Number) | | |  |  |
|  |  | Health post…………(Number) | | |  |  |
|  |  | Health Center…………(Number) | | |  |  |
|  |  | Primary Hospital……(Number) | | |  |  |
|  |  | General Hospital……(Number) | | |  |  |
|  |  | Specialized Hospital…...(Number) | | |  |  |
|  |  | Other…………(Number) | | |  |  |
|  |  | Total death………(Number) | | |  |  |
| 322 | Did the health facility take FBDA (**Annex 5A: Facility Based Data Abstraction)** for maternal death occurred in health facility (**?** | 1= Yes  2= No | | | If 2→326 |  |
| 323 | If yes Q322, numbers of maternal death conducted facility based data abstraction completed in 2009E.C? (See the document) | Within 24 hour…………(Number) | | |  |  |
|  |  | From 24 hours to 1 week … | | |  |  |
|  |  | Greater than 1 week …...(Number) | | |  |  |
| 324 | Is there any missed variable in the filled FBDA format for maternal death?(Observation ) | 1= Yes  2= No | | | If 2 →326 |  |
| 325 | If yes Q324, how many FBDA for maternal death formats are with missed variable in 2009 E.C? | ………(Number) | | |  |  |
| 326 | Did the health facility receive verbal autopsy (**Annex 4A: verbal autopsy)** for maternal death from health post?) **(for health center or primary hospital only )** | 1= Yes  2= No | | | If 2→ 330 |  |
| 327 | If Yes Q326, Numbers of verbal autopsy received for maternal death in 2009 E.C? **(for health center or primary hospital only )** (See the document) | Within 3-4 week……….(Number) | | |  |  |
|  |  | Greater than 4 week…….(Number | | |  |  |
| 328 | If Yes Q326 is there any missed variable in the filled verbal autopsy format for maternal death? (Observation ) | 1= Yes  2= No | | | If 2→330 |  |
| 329 | If Yes Q328, how many formats are with missed variable in 2009 E.C? | ………(Number ) | | |  |  |
| 330 | Did the health facility reviews for maternal deaths occurred? **(See document**) | 1= Yes  2= No | | |  |  |
| 331 | If Yes Q330, Numbers of conducted death reviewing for maternal death in 2009 E.C? (After verbal autopsy and facility based data abstraction ) **(See document**) | Within one week………(Number) | | |  |  |
|  |  | Greater than one week…(Number) | | |  |  |
| 332 | Was there perinatal death in 2009 E.C in your cluster? | 1= Yes  2= No | | |  |  |
| 333 | If yes Q332, number of perinatal death in 2009 E.C? (See the document) | Still birth ……….(Number) | | |  |  |
|  |  | Early Neonatal……….(Number) | | |  |  |
|  |  | Late Neonatal……….(Number) | | |  |  |
| 334 | If yes Q No 332, number of perinatal death in 2009 E.C? | Community……….(Number) | | |  |  |
|  |  | Health facility……….(Number) | | |  |  |
| 335 | Did the health facility take FBDA (**Annex 5B**: FBDA) for perinatal death occurred in health facility? | 1= Yes  2= No | | | If 2→339 |  |
| 336 | If yes Q335, numbers of perinatal death were facility based data abstraction completed in 2009 E.C? (See the document) | Within 24 hour……….(Number) | | |  |  |
|  |  | From 24 hours to 1 week ……… | | |  |  |
|  |  | Greater than 1 week …...(Number) | | |  |  |
| 337 | If yes Q335, is there any missed variable in the filled FBDA format for perinatal death?(Observation ) | 1= Yes  2= No | | | If 2→339 |  |
| 338 | If yes Q337, how many FBDA for perinatal formats are with missed variable in 2009 E.C? | ………(Number) | | |  |  |
| 339 | Did the health facility receive verbal autopsy **(Annex 4B: verbal autopsy)** for community based perinatal death from health post?  (**For Primary hospital and HC only**) **(See the document)** | 1= Yes  2= No | | | If 2→343 |  |
| 340 | If yes Q339, Numbers of perinatal death received verbal autopsy in 2009 E.C? **(For health center and primary hospital)** | within one week……….(Number) | | |  |  |
|  |  | Greater than one week....(Number) | | |  |  |
| 341 | If yes Q339, is there any missed variable in the filled verbal autopsy format for perinatal death? (Observation ) | 1= Yes  2= No | | | If 2→345 |  |
| 342 | If yes Q341, how many formats for community perinatal verbal autopsy are with missed variable in 2009 E.C? | ………(Number) | | |  |  |
| 343 | Did the health facility reviews for perinatal deaths? **(See the document)** | 1= Yes  2= No | | | If 2→345 |  |
| 344 | If Yes Q343, Numbers of perinatal death conducted death reviewing in 2009 E.C? (After verbal autopsy and facility based data abstraction completed) **(See the document)** | within one week……….(Number) | | |  |  |
|  |  | Greater than one week…(Number) | | |  |  |
| **Communication /feedback related questions** | | | | | | |
| 345 | Was the health facility send feedback to respective health center or primary hospital? | | | 1= Yes  2= No | If 2→347 |  |
| 346 | If yes Q345, how many feedback in 2009 E.C? (see document) | | | ………(Number) |  |  |
| 347 | Was the respective health facility got hospital’s feedback for hospital based death? *(* **for health center and p. hospital)** | | | 1= Yes  2= No | If 2→350 |  |
| 348 | **If yes Q347**, how many feedback in 2009 E.C? (see document) | | | ………(Number) |  |  |
| 349 | If Q 347 yes, was the health facility triangulating the hospital’s feedback during reviewing? ***(*for health center and primary hospital)** | | | 1= Yes  2= No |  |  |
| 350 | Is there proper document handling? (From observation of all perinatal and maternal reviewed) | | | 1= Yes  2= No |  |  |
| **Response related questions** | | | | | | |
| 351 | If yes Q330, Was action plan developed for reviewed maternal deaths? | | | 1= Yes  2= No | If 2→353 |  |
| 352 | If yes Q351, how many action plans developed in 2009 E.C? (see document) | | | ………(Number) |  |  |
| 353 | If yes Q343, was action plan developed for reviewed perinatal deaths? | | 1= Yes  2= No | | If 2→355 |  |
| 354 | If yes Q353, how many action plans developed in 2009 E.C? (see document) | | | ………(Number) |  |  |
| 355 | Did the health facility made a discussion about the main cause of the death with its staff members? (See minute) | | | 1= Yes  2= No |  |  |
| 356 | If yes Q355, What measures have been taken in response to maternal/perinatal?  (See minute) | | | 1= On job training was given  2=Availability of medical supply was improved  3=Constructing maternity waiting room  4= Arrangement of layout of the health facility  5= Corrective measures like punishment was taken  6=Others (Specify………………) |  |  |
| 357 | Did the health center/primary hospital made a discussion about the main cause of the death with WDA members in your catchment so as to prevent further death? | | | 1= Yes  2= No | If 2→359 |  |
| 358 | If yes Q357, what issues were raised? (Multiple responses are possible) (see document) | | | 1= Discussion on the importance of ANC service  2= Discussion on the importance of skilled delivery  3= Discussion on the importance of PNC service  4= Discussion on the importance of birth preparedness and complication readiness  5=Discussion on pregnancy related danger signs  6= Discussion on perinatal period danger signs  7=Other………...........................) |  |  |

| **Part II: Information to be collected from surveillance focal person** | | | | |
| --- | --- | --- | --- | --- |
| **General information related questions** | | | | |
| 359 | Is the health facility’s surveillance focal person trained for MPNDSR? | 1= Yes  2= No |  |  |
| 360 | Work experience of the surveillance focal person in complete years? | (Year----------) |  |  |
| 361 | Is the health facility incorporated maternal and perinatal death as part of its epidemic preparedness and response plan?(See document) | 1= Yes  2= No |  |  |
| 362 | Do you have uninterrupted registering of pregnant mothers? (See the document) | 1= Yes  2= No |  |  |
| 363 | Does the PHEM focal person communicate with respective HEW to check the survival status of the neonate at 7^th^ and 28 days? (see document) | 1= Yes  2= No | If 2→365 |  |
| 364 | If Q363 yes, how do you communicate? | 1= Telephone communication  2= written feedback  3= Other(specify----------------) |  |  |
| **Notification related questions** | | | | |
| 365 | Did health posts notify maternal death to health facility informally? (**For Primary hospital and HC only**) | 1= Yes  2= No |  |  |
| 366 | Did health posts notify maternal death to health facility formally **(Annex 1A)?** (**For Primary hospital and HC only**) | 1= Yes  2= No | If 2→368 |  |
| 367 | If Q366 yes, number of formally notified maternal death from health posts in 2009 E.C? (see document) | within 24 hrs………(Number ) |  |  |
|  |  | greater than 24hrs ………(Number ) |  |  |
| 368 | Did you notify maternal death to district health office/RHB? | 1= Yes  2= No |  |  |
| 369 | Did you notify formally **(Annex 1A)** institutional maternal death to head of health facility? | 1= Yes  2= No | If 2→373 |  |
| 370 | If Q369 yes, number of formally notified maternal death to head of health facility in 2009 E.C? (see document) | within 24 hrs.………(Number ) |  |  |
|  |  | greater than 24hrs ………(Number ) |  |  |
| 371 | If Q369 yes, is there any missed variable in the filled notification format for maternal death? (Observation) | 1= Yes  2= No | If 2→373 |  |
| 372 | If Q 371yes, how many formats are with missed variable in 2009 E.C? | ………(Number ) |  |  |
| 373 | Did health posts notify perinatal death to health facility informally? (**For Primary hospital and HC only**) | 1= Yes  2= No |  |  |
| 374 | Did health posts notify community perinatal death to health facility formally**(Annex 1B)**?(**For Primary hospital and HC only**) | 1= Yes  2= No | If 2→376 |  |
| 375 | If Q 374 yes, number of formally notified perinatal death in 2009 E.C?  **(Annex 1B)** | within 24 hrs………(Number ) |  |  |
|  |  | greater than 24 hrs………(Number ) |  |  |
| 376 | Did you notify perinatal death to district health office/RHB? | 1= Yes  2= No |  |  |
| 377 | Did you notify formally **(Annex 1B)** perinatal death to head of health facility? | 1= Yes  2= No | If 2→381 |  |
| 378 | If Q 377 yes, number of formally notified perinatal death to head of health facility in 2009 E.C?  **(Annex 1B)** | within 24 hrs.………(Number ) |  |  |
|  |  | greater than 24 hrs.………(Number ) |  |  |
| 379 | If Q 377 yes, is there any missed variable in the filled notification format for community perinatal death? (Observation) | 1= Yes  2= No |  |  |
| 380 | If Q 379 yes, how many formats are with missed variable in 2009 E.C? | ………(Number) |  |  |
| **Report related questions** | | | | |
| 381 | Did you report weekly PHEM report to district health office/RHB? | 1= Yes  2= No | If 2→383 |  |
| 382 | If Q381 yes, number of weekly reports sent to district office/RHB in 2009 E.C? **(see copy)** | ………(Number) |  |  |
| 383 | Did the health facility have timely surveillance report (Sunday or Monday)? (See the document) | 1= Yes  2= No | If 2→385 |  |
| 384 | If Q383 yes, number of timely reported? (see document) | ………(Number) |  |  |
| 385 | Did you send weekly zero report regarding maternal death to district health office/RHB? | 1= Yes  2= No | If 2→387 |  |
| 386 | If Q385 yes, Number of zero weekly reports in 2009 E.C? | ……….(Number) |  |  |
| 387 | Did you send weekly zero report regarding perinatal death to district health office/RHB? | 1= Yes  2= No | If 2→389 |  |
| 388 | If Q387 yes, Number of zero weekly reports in 2009 E.C? | …….(Number) |  |  |
| 389 | Did you send summary maternal death report **(Annex 6A: Summary maternal Death Report)** to district health office/RHB and respective health facility? | 1= Yes  2= No | If 2→394 |  |
| 390 | If Q389 yes, numbers summary maternal death report send in 2009 E.C? | TRHB…….(Number) |  |  |
|  |  | District…….(Number) |  |  |
|  |  | Respective facility…….(Number) |  |  |
| 391 | If Q No 390 is yes, after how many weeks did you send summary maternal death report in 2009 E.C? | with one week…….(Number) |  |  |
|  |  | Greater than one week…….(Number) |  |  |
| 392 | If Q389 yes, is there any missed variable in summary maternal death report? (Observation) | 1= Yes  2= No |  |  |
| 393 | If Q 392 yes, how many formats are with missed variable in 2009 E.C? | …….(Number) |  |  |
| 394 | Did you send summary perinatal death report (Summary perinatal Death report) to district health/TRHB and respective health facility? | 1= Yes  2= No | If 2→399 |  |
| 395 | If Q389 yes, numbers summary perinatal death report send in 2009 E.C? | TRHB…….(Number) |  |  |
|  |  | District…….(Number) |  |  |
|  |  | Respective facility…….(Number) |  |  |
| 396 | If Q No 394 is yes, after how many weeks did you summary perinatal death report in 2009 E.C? | Within one week…….(Number) |  |  |
|  |  | Greater than one week…….(Number) |  |  |
| 397 | If Q394 yes, is there any missed variable in summary perinatal death report? (Observation) | 1= Yes  2= No |  |  |
| 398 | If Q 392 yes, how many formats are with missed variable in 2009 E.C? | …….(Number) |  |  |
| **Registration and format materials related questions** | | | | |
| 399 | Availability of rumor logbook that document maternal and perinatal death? (See document) | 1= Yes  2= No |  |  |
| 401 | Check the availability of formats/annexes and circle all available formats | 1.Maternal Death Notification format  2.Perinatal Death Notification format  3.Maternal verbal Autopsy format  4. prenatal verbal Autopsy format  5.Maternal Death Facility Based Abstraction format  6. prenatal Death Facility Based Abstraction format  7.Maternal Death Case Based Report format  8.Perinatal Death Case Based Report format  9.Action Plan format  10. Referral and Neonatal Tracking tool (Annex 8A and 8B) |  |  |
| 402 | Availability of MPNDSR guideline?  (See document) | 1= Yes  2= No |  |  |
| 403 | Availability of report timeliness tracking mechanism? (See document) | 1= Yes  2= No |  |  |
| 404 | Availability of report completeness tracking mechanism? (See document) | 1= Yes  2= No |  |  |
| 405 | Availability of maternal death registration?  (See document) | 1= Yes  2= No |  |  |
| 406 | Availability of perinatal death registration?  (See document) | 1= Yes  2= No |  |  |
| 407 | Availability of surveillance weekly report format?(See document) | 1= Yes  2= No |  |  |

**I thank you!**

**ናይ ጥዕና ትካላት መሕትት**

**ቅጥዒ ሓበሬታ መውሃቢን ዉዕል ስምምዕነትን**

**መእተዊ፡-**

ጥዕና ይሃበለይ! ሽመይ………………………………ይበሃል:: ኢንስቲትዩት ምርምር ጥዕና ትግራይ ምስ ቢሮ ሓለዋ ጥዕና ክልል ትግራይን UNFPAን ብምትሕግጋዝ ንትግበራ ፈተሸ ሞት ኣዴታትን ሕንጦታትን ምላሽ ምሃብን ፕሮግራም ንዘካይድዎ ናይ መፅናዕቲ ሓበሬታ ንምእካብ እየ መፂአ፡፡ እዚ መፅናዕቲ ብትግራይ ደረጃ ኣብ ዝተመረፃ 22 ወረዳታት ኣብ ዝርከባ ጥዕና ትካላት ዝካየድ እንትኸውን ትካልኩም/ክን ድማ ካብተን ዝተመረፃ ትካላት ሓንቲ እያ፡፡ ኣብዚ መፅናዕቲ ዝሳተፉ ድማ ሓለፍቲ ትካላት፡ ናይ ሰርቨይላንስ ኪኢላታትን ጥሙር ጥዕና ሰራሕተኛታትን እዮም። ተሳትፎኹም ነዚ ኣብ ፈተሸ ሞት ኣዴታትን ሕንጦታትን ምላሽ ምሃብን አመልኪቱ ንዝካየድ መፅናዕቲ ብጣዕሚ አገዳሲ እዩ፡፡ እዚ ቃለ መሕተት ንኣስታት ሓደ ሰዓት ዝወስድ እንትኸውን ሽሞም/ምክን ኣይምዝገብን፡፡ እትህቡና/ባና ሓበሬታ ምስጢራዊ እዩ፡፡ ብምስታፍኩም/ኽን ዘምፅአልክን/ኩም ሳዕቤን የብሉን፡፡ምስታፍኩም/ክን አብ ድልየት ዝተመስረተ እትኸውን ክትምልስዎ/ኦ ዘይትድልይዎ/ኦ ሕቶ ምዝላል ትኽእሉ/ላ ኢኹም/ክን፡፡ኣብዚ መፅናቲ ንምስታፍ ፍቓደኛ ዲኹም/ኽን?

- ኣይኮንኩን…………….(“የቐንየለይ፡፡” ብምባል እዚ ቓለ-መሕተት የቓርፁ/ፃ::)
- እወ………………. (“የቐንየለይ::” ብምባል ንተሓታቲ ድሕሪ ምፍራም ቃለ መሕተቶም/ን ይቐፅሉ/ላ፡፡)

ፌርማ ተሓታቲ፡--------------------------------

ሓላፍነት ተሓታቲ፡-------------------------------

ሽም ሓታቲ ----------------------------- ፌርማ----------------ዕለት----------------

መፍለጢ ቑፅሪ መሕትት------------------

ነዚ ዘረጋገፀ ተቆፃፃሪ ሽም ------------------------ፌርማ---------------- ዕለት----------------

**ኢንስቲትዩት ምርምር ጥዕና ትግራይ**

**ብርኪ ትግበራ ፈተሸ ሞት ኣዴታትን ሕንጦታትን ምላሽ ምሃብን ኣብ ትግራይ፣ ሰሜን ኢትዮጵያ።**

**ክፍሊII: ናይ ጥዕና ትካላት መሕትት (ክፍሊ ሓደ ካብ ሓላፊ ጥዕና ትካል ዝእከብ)**

| **ሓፈሻዊ ሓበሬታ ካብ ሓለፍቲ ትካል** | | | **ዝለል** | **ኮድ** |  |
| --- | --- | --- | --- | --- | --- |
| 301 | ሽም ወረዳ | ………………… |  |  |  |
| 302 | ሽም ትካል ጥዕና | ………………… |  |  |  |
| 303 | ብርኪ ትካል ጥዕና? | 1= ጣብያ ጥዕና  2= መባእታዊ ሆስፒታል  3= ሓፈሻዊ ሆስፒታል  4= ሪፈራል ሆስፒታል |  |  |  |
| 304 | ዋንነት ትካል ጥዕና | 1= መንግስታዊ  2= ናይግሊ  3= ካሊእ (ጥቐስ………………) |  |  |  |
| 305 | በዝሒ ህዝቢ (ናይ 2010 ዓ.ም) | ጠቅላላ በዝሒ ህዝቢ…….  ተባ……ኣን……………..  ደቂ ኣንስትዮ 15-49 ዓመት…….  ትሕቲ 5 ዓመት……………….. |  |  |  |
| 306 | ናይ ክላስተር በዝሒ ጥ/ጥዕና ቤተሰብ ሰራሕተኛታት ?  **( ንጥዕና ጣብያን መባእታዊ ሆስፒታልን)** | ………….(ቁፅሪ) |  |  |  |
| 307 | ናይ ክላስተር በዝሒ ልምዓት ጉጅለ **(ንጥዕና ጣብያን መባእታዊ ሆስፒታልን)** | ………….(ቁፅሪ) |  |  |  |
| 308 | ጥዕና ትካልኩም MPNDSR/RRT ኣጣይሹ ዶ? | 1=እወ  2= ኣይፋሉን | መልሲ2→310 |  |  |
| 309 | መልስኹም/ኽን እወ እንተኾይኑ ኣባላት እቲ ኮሚቴ ካበይ ካበይ ተጣይሾም?  **(ካብ ሓደ ንላዕሊ መልሲ ይካኣል እዩ)** | 1=ካብ ምምሕዳር (ድጋፍ ወሃብቲ) ስታፍ  2=ካብ ሰብ ሞያጥዕና  3=ካብ ተወከልቲ ሕ/ሰብ  4= ካሊእ እንተሊዩ ጥቀሱ ---------- |  |  |  |
| 310 | በዝሒ ሰብ ሞያ ጥዕና MPNDSR ዝሰልጠኑ? **(ደኩመንት ይፈትሹ)** | መዋልዳን……..(ቁፅሪ)  ነርስ………….(ቁፅሪ)  ጥዕና መኮነን………..(ቁፅሪ)  በዓልሞያድንገተኛ መጥባሕቲ …  ሓፈሻዊ ሓኪም ………(ቁፅሪ)  ናይ ማህፀን ሓኪም ስፔሻሊስት……  ካሊእ እንተልዩ ይግለፁ…….(ቁፅሪ) |  |  |  |
| 311 | ናይዚ ጥዕና ትካል መራሒ ብዛዕባ MPNDSR ሰልጢኑ ዶ? | 1= እወ  2= ኣይፋሉን |  |  |  |
| 312 | ናይዚ ጥዕና ትካል መራሒ ስራሕ ልምዲ? (ብሙሉኣ ዓመት) | (ዓመት---------) |  |  |  |
| 313 | ናይ MPNDSR ኮሚቴ ኣቦ ወንበር ዓይነት ሞይኡ? | ------------------- |  |  |  |
| 314 | ናይ MPNDSR ኮሚቴ ኣቦ መንበር ስራሕ ሓላፍነት? | ------------------- |  |  |  |
| 315 | ናይ MPNDSR ኮሚቴ ኣቦ መንበር ንኽንደይ ዓመት መሪሑዎ? | ---------- (ብዓመት) |  |  |  |
| 316 | ኣብ 2009 ዓም በዝሒ ዝተኻየዱ MPNDSR ኮሚቴ ኣኼባ? **(ካብቃለኣኼባ ረአ**) | ……….(ቁፅሪ) |  |  |  |
| 317 | ኣብ 2009 ዓም ብዛዕባ ሞት ኣዴታትን ሕንጦታትን ኣጀንዳ ዝሓዘ ናይ ክላስተር ኣኼባ ዝካየዱ በዝሒ?  **(ካብ ቃለ ኣኼባ ረአ )** | ……….(ቁፅሪ) |  |  |  |
| 318 | ብዛዕባ ሞት ኣዶን ሕንጦን ህዝባዊ ኮንፈረንስ ይካየድ ዶ? | 1= እወ  2= ኣይፋል | መልሲ2→320 |  |  |
| 319 | ንሕቶ ቁ 318 መልስኹም እወ እንተኾይኑ ኣብ 2009 ዓ/ም ብዛዕባ ሞት ኣዶን ሕንጦን ክንደይ ግዜ ናይ ህዝቢ ኮንፈረንስ ተኻይዱ? **(ደኩመንት ይፈትሹ)** | ……….(ቁፅሪ) |  |  |  |
| **ምላሽ ምሃብ ዝምልከት መሕትት** | | | | |  |
| 320 | ኣብ 2009 ዓ/ም ኣብ ክላስተርኩም ሞት ኣዶ ኣጋጢሙኩም ዶ? | 1= እወ  2= ኣይፋሉን |  |  |  |
| 321 | ንሕቶ ቁ 320 መልስኹም እወ እንተኾይኑ ኣብ 2009 ዓ/ም በዝሒ ዝሞታ ብመሰረት ዝሞታሉ ቦታ የቐምጡ? **(ደኩመንት ረኣዩ)** | ኣብ ገዛ …………….(ቁፅሪ) |  |  |  |
|  |  | ኣብ መንገዲ ………….(ቁፅሪ) |  |  |  |
|  |  | ኣብ ጥዕና ኬላ ………….(ቁፅሪ) |  |  |  |
|  |  | ኣብ ጥዕና ጣብያ ………..(ቁፅሪ) |  |  |  |
|  |  | ኣብ መባእታዊ ሆስፒታል….(ቁፅሪ) |  |  |  |
|  |  | ኣብ ሓፈሻዊ ሆስፒታል…….(ቁፅሪ) |  |  |  |
|  |  | ኣብ ሪፈራል ሆስፒታል…….(ቁፅሪ) |  |  |  |
|  |  | ካሊእ እንተልዩ ይግለፁ.….(ቁፅሪ) |  |  |  |
|  |  | ተቅላላ በዝሒ ዝሞታ……….(ቁፅሪ) |  |  |  |
| 322 | ኣብ ጥዕና ትካልኩም ንዘጋጠሞ ሞት ኣዶ ካብ ጥዕና ትካል ዝምላእ ሓበሬታ ምኽንያት ሞት (**Annex 5A**: Facility Based Data Abstruction-(FBDA)) ትወስዱለን ዶ? | 1= እወ  2= ኣይፋል | መልሲ2→326 |  |  |
| 323 | ንሕቶ ቁ 322 መልስኹም እወ እንተኾይኑ ኣብ 2009 ዓ/ም ክንደይ ዝሞታ ኣዴታት FBDA ተወሲዱለን?  **(ዝተመለአ ፎርም ብምረኣይ ይረጋገፅ)** | ኣብ ውሽጢ 24 ሰዓት……. (ቁፅሪ) |  |  |  |
|  |  | 24 ሰዓት ክሳብ 1 ሰሙን …(ቁፅሪ) |  |  |  |
|  |  | ድሕሪ 1 ሰሙን ……….. (ቁፅሪ) |  |  |  |
| 324 | ንሕቶ ቁ **322** መልስኹም እወ እንተኾይኑ ኣበቲ ቕጥዒ ከይተመለአ ዝተረፈ ሓበሬታ ዘለዎ ቕጥዒ ኣሎ ዶ?  **(ምጉዳል ሙሉእነት ቕጥዕታት ረአ)** | 1= እወ  2= ኣይፋል | መልሲ2→326 |  |  |
| 325 | ንሕቶ ቁ 324 መልስኹም/ክን እወ እንተኾይኑ ናይ 2009 ዓ/ም ክንደይ ምሉእነት ዘይብሎም ቕጥዕታት ኣለው? | ………(ቁፅሪ) |  |  |  |
| 326 | ጥዕና ትካልኩም ናይ ምኽንያት ሞት ምርመራ ድሕሪ ሞት ኣዴታት (Annex 4A: verbal autopsy) ሓበሬታ ካብ ታሕቲ/ ጥ/ኬላ ይቅበል ዶ? **(ንጥዕና ጣብያን መባእታዊ ሆስፒታልን ጥራሕ ዝሕተት)** | 1= እወ  2= ኣይፋሉን | መልሲ2→ 330 |  |  |
| 327 | ንሕቶ ቁ 326 መልስኩም/ክን እወ እንተኾይኑ ኣብ 2009 ዓ/ም እተን ዝሞታ ኣዴታት ድሕሪ ክንደይ ሰሙን verbal autopsy ተወሲዱለን? **(ንጥዕና ጣብያን መባእታዊ ሆስፒታልን ጥራሕ ዝሕተት)**  **(ዝተመለአ ፎርም ብምረኣይ ይረጋገፅ)** | ኣብ ዉሽጢ 3-4 ሰሙን….. (ቁፅሪ) |  |  |  |
|  |  | ድሕሪ 4 ሰሙን……… (ቁፅሪ) |  |  |  |
| 328 | ንሕቶ **ቁ 326** መልስኹም/ክን እወ እንተኾይኑ ኣብቲ ቕጥዒ ከይተመለአ ዝተረፈ ሓበሬታ ዘለዎ ቕጥዒ ኣሎ ዶ?  **(ምጉዳል ሙሉእነት ቕጥዕታት ረአ)** | 1= እወ  2= ኣይፋሉን | መልሲ2→330 |  |  |
| 329 | ንሕቶ **ቁ 328** መልስኽም/ክን እወ እንተኾይኑ ኣብ 2009 ዓ/ም ክንደይ ምሉእነት ዘይብሎም ቕጥዕታት ኣለው? | ………(ቁፅሪ) |  |  |  |
| 330 | ጥዕና ትካልኩም ገምጋም ምኽንያት ሞት ሞት ኣዶ (Annex 6A death reviewing) የካይድ ዶ? | 1= እወ  2= ኣይፋሉን | መልሲ2→332 |  |  |
| 331 | ንሕቶ ቁ 330 መልስኹም/ክን እወ እንተኾይኑ ኣብ 2009ዓ/ም ክንደይ ገምጋማ ሞት ኣዶ ኣካይድኩም? **(ድሕሪ ምክንያት ሞት ኣዶ ካብ ትካልን ገዛን ምስ ተኣከበ) (ደኩመንት ይርኣዩ)** | ኣብ ዉሽጢ ሓደ ሰሙን……(ቁፅሪ) |  |  |  |
|  |  | ድሕሪ ሰሙን………(ቁፅሪ) |  |  |  |
| 332 | ኣብ 2009 ዓ/ም ሞይቶም ዝተወለዱ ወይ ምስ ተወለዱ ዝሞቱ ሕንጦታት ኣብ ክላስተርኩም ኣለውኹም ዶ? | 1= እወ  2= ኣይፋሉን |  |  |  |
| 333 | ንሕቶ ቁ 332 መልስኹም/ክን እወ እንተኾይኑ ኣብ 2009ዓ/ም ክንደይ ሞይቶም ዝተወለዱን ምስ ተወለዱ ዝሞቱን ሕንጦታት ኣለውኹም?  **(ደክመንት ይፈትሹ)** | በዚሒ ሞይቶም ዝተወለዱ…… |  |  |  |
|  |  | በዚሒ ኣብ ውሽጢ 7 መዓልቲ ድ/ወሊድ ዝሞቱ….. |  |  |  |
|  |  | በዚሒ ካብ 8 - 28 መዓልቲ ዝሞቱ……….. |  |  |  |
| 334 | ንሕቶ **ቁፅሪ 332** መልስኹም/ክን እወ እንተኾይኑ ኣብ 2009 ዓ/ም በዝሕን ቦታን ይገለፅ? | ኣብ ገዛ……….(ብቁፅሪ) |  |  |  |
|  |  | ኣብ ትካል ጥዕና………(ብቁፅሪ) |  |  |  |
| 335 | ኣብ ጥዕና ትካልኩም ሞይቶም ዝተወለዱን ምስ ተወለዱ ዝሞቱን ሕንጦታት ብብረኪ ጥዕና ትካል ዝውሰድ ሓበሬታ ምክንያት ሞት (**Annex 5B**: FBDA) ትወስዱሎምዶ? | 1= እወ  2= ኣይፋሉን | መልሲ2→339 |  |  |
| 336 | ንሕቶ ቁ 335 መልስኹም/ኽን እወ እንተኾይኑ ኣብ 2009 ዓ/ም ክንደይ FBDA ተወሲዱ? **(ዝተመለአ ፎርም ብምረኣይ ይረጋገፅ)** | ኣብ ውሽጢ 24 ሰዓት……. (ቁፅሪ) |  |  |  |
|  |  | >24 ሰዓት ክሳብ 1 ሰሙን.... (ቁፅሪ) |  |  |  |
|  |  | ድሕሪ 1 ሰሙን ……….. (ቁፅሪ) |  |  |  |
| 337 | ንሕቶ **ቁ 335** መልስኹም/ክን እወ እንተኾይኑ ካብቶም ዝተመልኡ ቕጥዕታት FBDA ሙሉእነት ዝጎደሎም ቕጥዕታት ኣለዉ ዶ?  **(ምጉዳል ሙሉእነት ቕጥዕታት ረአ)** | 1= እወ  2= ኣይፋሉን | መልሲ2→339 |  |  |
| 338 | ንሕቶ ቁ 337 መልስኹም/ኽን እወ እንተኾይኑ ኣብ 2009 ዓ/ም ክንደይ FBDA ምሉእነት ዝጎደሎም ተረኪቦም? | ………(ቁፅሪ) |  |  |  |
| 339 | ጥዕና ትካልኩም ኣብ ሕ/ሰብ ሞይቶም ንዝተወለዱን ምስ ተወለዱ ንዝሞቱን ሕንጦታት ቕጥዒ ሓበሬታ ምክንያት ሞት (**Annex 4B**: verbal autopsy) ካብ ጥ/ኬላ ይቕበል ዶ?  **(ንጥዕና ጣብያን መባእታዊ ሆስፒታልን**  **ጥራሕ ዝእከብ ሓበሬታ)** (**ደኩመንት ይርኣዩ**) | 1= እወ  2= ኣይፋሉን | መልሲ2→343 |  |  |
| 340 | ንሕቶ **ቁ 339** መልስኹም/ክን እወ እንተኾይኑ ኣብ 2009 ዓ/ም እቶም ኣብ ሕ/ሰብ ሞይቶም ዝተወለዱን ምስ ተወለዱ ዝሞቱን ሕንጦታት ድሕሪ ክንደይ ሰሙን verbal autopsy ተወሲዱሎም?  **(ዝተመለአ ፎርም ብምረኣይ ይረጋገፅ)** | ኣብ ዉሽጢ ሓደ ሰሙን……(ቁፅሪ) |  |  |  |
|  |  | ድሕሪ 1 ሰሙን…… (ቁፅሪ) |  |  |  |
| 341 | ንሕቶ **ቁ 339** መልስኹም/ክን እወ እንተኾይኑ ካብቶም ዝተመልኡ ቕጥዕታት verbal autopsy ሙሉእነት ዝጎደሎም ቕጥዕታት ኣሎ ዶ?  **(ምጉዳል ሙሉእነት ቕጥዕታት ረአ)** | 1= እወ  2= ኣይፋል | መልሲ2→343 |  |  |
| 342 | ንሕቶ ቁ 341 መልስኹም/ኽን እወ እንተኾይኑ ኣብ 2009 ዓ/ም ክንደይ ቕጥዕታት ሙሉእነት ዝጎደሎም ኣለዉ? | ………(ቁፅሪ) |  |  |  |
| 343 | አብ ጥዕና ትካልኩም ገምጋም ምክንያት ሞት (**Annex 6B**: death reviewing) ሞይቶም ንዝተወለዱን ምስ ተወለዱ ንዝሞቱን ሕንጦታት ይካየድ ዶ? **(ድሕሪ ሓበሬታ ሞት ምስ ተኣከበ)** | 1= እወ  2= ኣይፋሉን | መልሲ2→345 |  |  |
| 344 | ንሕቶ ቁ 343 መልስኹም/ኽን እወ እንተኾይኑ ኣብ 2009 ዓ/ም ክንደይ ሞይቶም ንዝተወለዱን ምስ ተወለዱ ንዝሞቱን ሕንጦታት (death reviewing) ተገይርሎም?  **(ድሕሪ ሓበሬታ ሞት ምስ ተኣከበ)** | ኣብ ዉሽጢ ሰሙን……(ቁፅሪ) |  |  |  |
|  |  | ድሕሪ ሓደ ሰሙን……(ቁፅሪ) |  |  |  |
| **ርክብ /ግብረ- መልሲ ዝምልከት መሕትት** | | | | |  |
| 345 | ኣብ ትካልኩም ዝተኣከበ ሓበሬታ ምክንያት ሞት ኣዴታትን ሕንጦታትን ናብ ዝምልከተን ጥዕና ጥካላት ግብረ-መልሲ ይለኣኽ ዶ? | 1= እወ  2= ኣይፍሉን | መልሲ2→347 |  |  |
| 346 | ንሕቶ ቁ 345 መልስኹም/ኽን እወ እንተኾይኑ ኣብ 2009 ዓ/ም ክንደይ ግብረ-መልሲ ተላኢኹ?  **(ደኩመንት ረኣዩ)** | ……….(ቁፅሪ) |  |  |  |
| 347 | ኣብ ሆስፒታል ንዘጋጠመ ሞት ኣዴታት ትካልኩም ዝተኣከበ ሓበሬታ ምክንያት ሞት ካብ ሆስፒታል ግብረ መልሲ ይቕበል ዶ ?  **(ንጥዕና ጣብያን መባእታዊ ሆስፒታልን ጥራሕ ዝእከብ ሓበሬታ)** | 1= እወ  2= ኣይፋሉን | መልሲ2→350 |  |  |
| 348 | ንሕቶ ቁ 347 መልስኹም/ኽን እወ እንተኾይኑ ኣብ 2009ዓ/ም ክንደይ ግብረ-መልሲ ተቀቢልኩም?  **(ደኩመንትረኣዩ)** | ……(ቁፅሪ) |  |  |  |
| 349 | ንሕቶ ቁ 347 መልስኹም/ኽን እወ እንተኾይኑ ትካልኩም ካብ ሆስፒታል ዝተቐበልኩሞ ግብረ-መልሲ ምስ ባዕልኹም ዝኣከብኩሞ ሓበሬታ ኣካቲትኩም ትግምግምዎ ዶ?  **(ንጥዕና ጣብያን መባእታዊ ሆስፒታልን**) | 1= እወ  2= ኣይፋሉን |  |  |  |
| 350 | አብ ትካልኩም እቲ ዝተኣከበ ሓበሬታ ቕጥዒ ብትክክል ተቐምጥዎ ዶ?  **(ብፅቡቅ ኩነታት ዝተቀመጠ ምኻኑ ረአ)** | 1= እወ  2= ኣይፋሉን |  |  |  |
| **ምላሽ ምሃብ ዝምልከት መሕትት** | | | | |  |
| 351 | ንሕቶ ቁ 330 መልስኹም/ኽን እወ እንተኾይኑ ብትካልኩም ንዝተገምገሙ ምኽንያት ሞት ኣዴታት መተግበሪ ትልሚ ተዳሊውሎም ዶ? | 1= እወ  2= ኣይፋሉን | መልሲ2→353 |  | |
| 352 | ንሕቶ ቁ 351 መልስኹም/ኽን እወ እንተኾይኑ ኣብ 2009 ዓ/ም ክንደይ ብዛዕባ ሞት ኣዴታት መተግበሪ ትልሚ ተዳሊዉ? **(ደኩመንት ይርኣዩ)** | ……(ቁፅሪ) |  |  | |
| 353 | ንሕቶ ቁ 343 መልስኹም/ኽን እወ እንተኾይኑ ብትካልኩም ንዝተገምገሙ ምክንያት ሞይቶም ንዝተወለዱን ምስ ተወለዱ ንዝሞቱን ሕንጦታት መተግበሪ ትልሚ ተዳሊውሎም ዶ? | 1= እወ  2= ኣይፋሉን | መልሲ2→355 |  | |
| 354 | ንሕቶ ቁ 353 መልስኹም/ኽን እወ እንተኾይኑ ኣብ 2009 ዓ/ም ክንደይ ብዛዕባ ሞት ሕንጦታት መተግበሪ ትልሚ ተዳሊዉ? **(ደኩመንት ይርኣዩ)** | ……(ቁፅሪ) |  |  | |
| 355 | ጥዕና ትካልኩም ብዛዕባ ዝተነፀሩ ቀንዲ ምኽንያታት ሞት ኣዴታትን ሕንጦታትን መሰረት ጌሩ ምስ ጥዕና ትካል ሰራሕተኛታት ብምምይያጥ መፍትሒ ሓሳባት ይወስድ ዶ? | 1= እወ  2= ኣይፋል | መልሲ2→357 |  | |
| 356 | ንሕቶ ቁ 355 መልስኹም/ክን ኣወ እንተኾይኑ እንታይ መፍትሕታት ተወሲዶም?  **(ካብ ሓደ ንላዕሊ መልሲ ይካኣል እዩ)** | 1= ኣብ ከይዲ ስራሕ ምምህሃር  2=ኣቕርቦት መድሓኒት ንመሳርሒታትን ተማሓዪሹ  3=መፅንሒ ክፍሊ ጥኑሳትን ሓራሳትን ተሰሪሑ  4= ናይቲ ትካል ጥዕና ኣቀማምጣ ተመዓራሪዩ  5= መስተኻኸሊ ስጉምቲታት ከም መቅፃዕቲ ዛኣመሰሉ ተወሲዶም  6=ካሊእ (ጥቐሱ…………………) |  |  | |
| 357 | ጥዕና ትካልኩም ብዛዕባ ቀንዲ ምኽንያት ሞት ኣዴታትን ሕንጦታትን ምስ ኣባላት ልምዓት ጉጅለ ምይይጥ ብምክያድ ተመሳሳሊ ሞት ንምክልኻል ምይይጥ የካይድ ዶ?  **(ንጥዕና ጣብያን መባእታዊ ሆስፒታል ጥራሕ**) | 1= እወ  2= ኣይፋል | መልሲ2→359 |  | |
| 358 | ንሕቶ ቁ 357 መልስኹም እወ እንተኾይኑ ፣እንታይ ሓሳባት ተላዒሎም?  **(ካብ ሓደ ንላዕሊ መልሲ ይካኣል እዩ)** | 1= ኣብ ጠቐሜታ ቅ/ወሊድ ግልጋሎት  2= ኣብ ጠቐሜታ ኣብ ጥዕና ትካል ወሊድ ግልጋሎት  3= ኣብ ጠቐሜታ ድ/ወሊድ ግልጋሎት  4=ኣብ ጠቐሜታ ምድላው ኣብ ጥንስን ድ/ወሊድን ክኽሰቱ ዝክእሉ ሓደገኛምልክታት  5= ኣብ ጥንስን ድ/ወሊድን ክኽሰቱ ዝክእሉ ሓደገኛ ምልክታት  6= ኣብ ሕንጦታት ክኽሰቱ ዝክእሉ ሓደገኛ ምልክታት  7=ካሊእ (ጥቐሱ…………………) |  |  | |

| **ክፍሊ II: ካብ ሰርቪላንስ ፎካል ፐርሰን ዝእከብ ሓበሬታ** | | | | | | | |
| --- | --- | --- | --- | --- | --- | --- | --- |
| **ሓፈሻዊ ሓበሬታ** | | | | | | | |
| 359 | ናይቲ ጥዕና ትካል ሰርቪላንስ ፎካል ፐርሰን MPNDSR ዝሰልጠነ/ት ድዩ/ያ? | | 1= እወ  2= ኣይፋል |  | | |  |
| 360 | ስራሕ ልምዲ ሰርቪላንስ ፎካል ፐርሰን ብምሉእ ዓመት | | ---------- (ብዓመት) |  | | |  |
| 361 | ጥዕና ትካልኩም ናይ ፈተሸን ምላሽ ምሃብን ሞት ኣዴታትን ሕንጦታትን (MPNDSR) ምስ ናይ ቅደመ መጠንቀቕታን ድልውነት ምላሽ ምሃብን ናይቲ ትካል ትልሚ ተኻቲቱ ዶ? **(ደኩመንት ይርኣዩ)** | | 1= እወ  2= ኣይፋል |  | | |  |
| 362 | ጥዕና ትካልኩም ናይ ክላስተሩ ዘይተቆራረፀ በቢግዝኡ ዝተመሓየሸ ምዝገባ ጥኑሳት የካይድ ዶ?  **(ንጥዕና ጣብያን መባእታዊ ሆስፒታልን ጥራሕ ዝእከብ ሓበሬታ) (ደኩመንት ይርኣዩ)** | | 1= እወ  2= ኣይፋል |  | | |  |
| 363 | ናይቲ ጥዕና ትካል ሰርቪላንስ ፎካል ፐርሰን ኣብ ጥዕና ትካል ዝተወለዱ ሕንጦታት ምስ ጥሙር ጥዕና ሰራሕተኛታት ሃለዋት ሕንጦታት ንምርግጋፅ ኣብ 7ይን 28 መዓልቲ ርክብ ይገብሩ ዶ? **(Annex 8B: ደኩመንት ይርኣዩ)** | | 1= እወ  2= ኣይፋል | መልሲ2→365 | | |  |
| 364 | ንሕቶ ቁ 363 መልስኹም/ኽን እወ ኣንተኾይኑ ብኸመይ ርክብ  ይገብር? | | 1= ቴለፎን  2= ብፅሑፍ  3= ካሊእ እንተልዩ ይግለፁ(-------------) |  | | |  |
| **ናይ ምልላይ ምፍላጥን ሞት ዝምልከት መሕትት** | | | | | | | |
| 365 | ጥዕና ኬላታት ሞት ኣዴታት  ናብ ጥዕና ትካልኩም ብዘይስሩዕ የፍልጣ ዶ?**(ንጥዕና ጣብያን መባእታዊ ሆስፒታልን ጥራሕ ዝእከብ ሓበሬታ)** | | 1= እወ  2= ኣይፋል |  | |  | |
| 366 | ጥዕና ኬላታት ሞት ኣዴታት  ናብ ጥዕና ትካልኩም ብስሩዕ ፎርማት የፍልጣኹም ዶ?**(Annex 1A: ንጥዕና ጣብያን መባእታዊ ሆስፒታልን ጥራሕ ዝእከብ ሓበሬታ) (ደኩመንት ይርኣዩ)** | | 1= እወ  2= ኣይፋል | መልሲ2→368 | |  | |
| 367 | ንሕቶ ቁ 366 መልስኹም/ክን እወ ኣንተኾይኑ ኣብ 2009 ዓ/ም ክንደይ ሞት ኣዴታት ብስሩዕ ፎርማት ኣፍሊጠናኹም? | | ኣብ ዉሽጢ 24 ሰዓት…… (ቁፅሪ) |  | |  | |
|  |  |  | ድሕሪ 24 ሰዓት…… (ቁፅሪ) |  | |  |  |
| 368 | ጥዕና ትካልኩም ሞት ኣዴታት ናብ ወረዳ/ክልል የፍልጥ/ሪፖርት ይገብር ዶ? | | 1= እወ  2= ኣይፋል |  | |  | |
| 369 | ጥዕና ትካልኩም ሞት ኣዴታት  ብስሩዕ ፎርማት ናብ ሓላፊ እዚ ትካል ተፍልጡ ዶ?**(Annex 1A: ደኩመንት ይርኣዩ)** | | 1= እወ  2= ኣይፋል | መልሲ2→373 | |  | |
| 370 | ንሕቶ ቁ 369 መልስኹም/ክን እወ ኣንተኾይኑ ኣብ 2009 ዓ/ም ክንደይ ሞት ኣዴታት ብስሩዕ ፎርማት ኣፍሊጥኩም? | | ኣብ ዉሽጢ 24 ሰዓት…… (ቁፅሪ) |  | |  | |
|  |  |  | ድሕሪ 24 ሰዓት…… (ቁፅሪ) |  |  |  |  |
| 371 | ቁ 369 መልስኹም/ክን እወ እንተኾይኑ ካብቶም ዝተልኣኹ መፍለጢ ቕጥዕታት ሙሉእነት ዝጎደሎም ቕጥዕታት ኣለዉ ዶ? **(ምጉዳል ሙሉእነት ቕጥዕታት ረአ)** | | 1= እወ  2= ኣይፋል |  | |  | |
| 372 | ንሕቶ ቁ 371 መልስኹም/ክን እወ እንተኾይኑ ኣብ 2009 ዓ/ም ክንደይ ምሉእነት ዘይብሎም ዘለዎ ቕጥዒታት መፍለጢ ሞት ኣዴታት ኣሎ? | | ……(ቁፅሪ) |  | |  | |
| 373 | ጥዕና ኬላታት ሞይቶም ንዝተወለዱን ምስ ተወለዱ ንዝሞቱን ሕንጦታት ናብ ጥዕና ትካልኩም ብዘይስሩዕ የፍልጣ ዶ? **(ንጥዕና ጣብያን መባእታዊ ሆስፒታልን ጥራሕ ዝእከብ ሓበሬታ)** | | 1= እወ  2= ኣይፋል |  | |  | |
| 374 | ጥዕና ኬላታት ኣብ ሕ/ሰብ ሞይቶም ንዝተወለዱን ምስ ተወለዱ ንዝሞቱን ሕንጦታት ናብ ጥዕና ትካልኩም ብስሩዕ ፎርማት የፍልጣ ዶ? **(Annex 1B: ካብ ጥዕና ጣብያን መባእታዊ ሆስፒታልን ጥራሕ ዝእከብ ሓበሬታ) (ደኩመንት ይርኣዩ)** | | 1= እወ  2= ኣይፋል | መልሲ2→376 | |  | |
| 375 | ንሕቶ ቁ 374 መልስኹም/ክን እወ እንተኾይኑ ኣብ 2009 ዓ/ም ክንደይ ሞይቶም ዝተወለዱን ምስ ተወለዱ ዝሞቱን ሕንጦታት ብስሩዕ ፎርማት ኣፍሊጠናኹም?  **(Annex 1B: ደኩመንት ይርኣዩ)** | | ኣብ 24 ሰዓት…… (ቁፅሪ) |  | |  | |
|  |  |  | ድሕሪ 24 ሰዓት…… (ቁፅሪ) |  |  |  |  |
| 376 | ጥዕና ትካልኩም ሞይቶም ንዝተወለዱን ምስ ተወለዱ ንዝሞቱን ሕንጦታት ናብ ወረዳ ሪፖርት ይግበር ዶ?  **(Annex 1B: ደኩመንት ይርኣዩ)** | | 1= እወ  2= ኣይፋል |  | |  | |
| 377 | ጥዕና ትካልኩም ሞይቶም ንዝተወለዱን ምስ ተወለዱ ንዝሞቱን ሕንጦታት ናብ ሓላፊ እዚ ትካል ብስሩዕ ፎርማት ተፍልጡ ዶ?**(Annex 1B: ደኩመንት ይርኣዩ)** | | 1= እወ  2= ኣይፋል | መልሲ2→381 | |  | |
| 378 | ንሕቶ ቁ 377 መልስኹም/ክን እወ ኣንተኾይኑ ኣብ 2009 ዓ/ም ክንደይ ሞይቶም ንዝተወለዱን ምስ ተወለዱ ንዝሞቱን ሕንጦታት ብስሩዕ ፎርማት ኣፍሊጥኩም? | | ኣብ ዉሽጢ 24 ሰዓት…… (ቁፅሪ) |  | |  | |
|  |  |  | ድሕሪ 24 ሰዓት…… (ቁፅሪ) |  |  |  |  |
| 379 | ቁ 377 መልስኹም/ክን እወ እንተኾይኑ ካብቶም ዝተለኣኩ መፍለጢ ቕጥዕታት ሙሉእነት ዝጎደሎም ቕጥዕታት ኣለዉ ዶ? **(ምጉዳል ሙሉእነት ቕጥዕታት ረአ)** | | 1= እወ  2= ኣይፋል |  | |  | |
| 380 | ንሕቶ ቁ 379 መልስኹም/ክን እወ እንተኾይኑ ኣብ 2009 ዓ/ም ክንደይ ምሉእነት ዘይብሎም ቕጥዒ መፍለጢ ሞት ኣዴታት ኣሎ? | | ……(ቁፅሪ) |  | |  | |
| **ሪፖርት ዝምልከት መሕትታት** | | | | | | | |
| 381 | ሰሙናዊ PHEM ሪፖርት ናብ ወረዳ/ክልል ሪፖርት ይግበር ዶ? | | 1= እወ  2= ኣይፋል | መልሲ2  →383 | |  | |
| 382 | ንሕቶ ቁ 381መልስኹም/ኽን እወ እንተኾይኑ ኣብ 2009 ዓ/ም ክንደይ ሰሙናዊ PHEM ሪፖርት ተላኢኹ? **(ደኩመንት ይርኣዩ)** | | ……(ቁፅሪ) |  | |  | |
| 383 | ሰሙናዊ PHEM ሪፖርት ናብ  ወረዳ/ክልል ብእዋኑ ይለኣኽ ዶ?  (ሰንበት ወይ ሶኑይ) **(ሰሙናዊ**  **እዋናውነት መካታተሊ ቅጥዒ ረአ**) | | 1= እወ  2= ኣይፋል | መልሲ2→385 | |  | |
| 384 | ንሕቶ ቁ 383 መልስኹም/ኽን እወ እንተኾይኑ ኣብ 2009 ዓ/ም ክንደይ ብእዋኑ ዝተለኣኸ ኣሎ? | | ……(ቁፅሪ) |  | |  | |
| 385 | ጥዕና ትካልኩም ሰሙናዊ ዜሮ ሪፖርት ሞት ኣዴታት ናብ ወረዳ/ክልል ይልእኽ ዶ? **(ደኩመንት ይርኣዩ)** | | 1= እወ  2= ኣይፋል | መልሲ2→387 | |  | |
| 386 | ንሕቶ ቁ 385 መልስኹም/ኽን እወ እንተኾይኑ ኣብ 2009 ዓ/ም ክንደይ ሰሙናዊ ዜሮ ሞት ኣዴታት ሪፖርት ተላኢኹ? **(ደኩመንት ይርኣዩ)** | | ……(ቁፅሪ) |  | |  | |
| 387 | ጥዕና ትካልኩም ሰሙናዊ ዜሮ ሪፖርት ሞይቶም ንዝተወለዱን ምስ ተወለዱ ንዝሞቱን ሕንጦታት ናብ ወረዳ/ክልል ይልእኽ ዶ? **(ደኩመንት ይርኣዩ)** | | 1= እወ  2= ኣይፋል | መልሲ2→389 | |  | |
| 388 | ንሕቶ ቁ 387 መልስኹም/ኽን እወ እንተኾይኑ ኣብ 2009 ዓ/ም ክንደይ ሰሙናዊ ዜሮ ሪፖርት ተላኢኹ?**(ደኩመንት ይርኣዩ)** | | ……(ቁፅሪ) |  | |  | |
| 389 | ጥዕና ትካልኩም ድሕሪ ገምጋም ምኽንያት ሞት ኣዶ መጠቓለሊ ሪፖርት **(Annex 6A: Summary maternal Death Report)** ናብ ወረዳ/ክልልን ዝምልከተን ጥዕና ትካልን **ት**ሰዱ ዶ? | | 1= እወ  2= ኣይፋል | መልሲ2→394 | |  | |
| 390 | ንሕቶ ቁ 389 መልስኹም/ኽን እወ እንተኾይኑ ኣብ 2009 ዓ/ም ክንደይ መጠቓለሊ ናይ ሞት ኣዴታት ሪፖርት ልኢኽኩም? **(ደኩመንት ይርኣዩ)** | | ናብ ክልል……(ቁፅሪ) |  | |  | |
|  |  |  | ናብ ወረዳ……(ቁፅሪ) |  |  |  |  |
|  |  |  | ናብ ዝምልከታ ጥዕና ትካል……(ቁፅሪ) |  |  |  |  |
| 391 | ንሕቶ ቁ 390 መልስኹም/ኽን እወ እንተኾይኑ ገምጋም ምኽንያት ሞት ኣዶ ድሕሪ ክንደይ ሰሙን ተላኢኹ?  (**Annex 6A: ደኩመንት ይርኣዩ**) | | ኣብ ዉሽጢ ሓደ ሰሙን……(ቁፅሪ) |  | |  | |
|  |  |  | ድሕሪ ሓደ ሰሙን……(ቁፅሪ) |  |  |  |  |
| 392 | ንሕቶ ቁ 389 መልስኹም/ክን እወ እንተኾይኑ ካብቶም ዝተለኣኹ መጠቓለሊ ናይ ሞት ኣዴታት ሪፖርት **(Annex 6A: Summary maternal Death Report)** ሙሉእነት ዝጎደሎም ቕጥዕታት ኣለዉ ዶ? **(ምጉዳል ሙሉእነት ቕጥዕታት ረአ)** | | 1= እወ  2= ኣይፋል | መልሲ2→394 | |  | |
| 393 | ንሕቶ ቁ 392 መልስኹም/ክን እወ እንተኾይኑ ኣብ 2009 ዓ/ም ክንደይ ፎርማት ምሉእነት ዘይብሎም መጠቓለሊ ናይ ሞት ኣዴታት ሪፖርት ኣሎ? **(See Annex 6A: Summary maternal Death Report)** | | ……(ቁፅሪ) |  | |  | |
| 394 | ጥዕና ትካልኹም ድሕሪ ገምጋም ምኽንያት ሞይቶም ንዝተወለዱን ምስ ተወለዱ ንዝሞቱን ሕንጦታት መጠቓለሊ ሪፖርት (Summary perinatal Death Report) ናብ ወረዳ/ክልል ትሰዱ ዶ? | | 1= እወ  2= ኣይፋል | መልሲ2→399 | |  | |
| 395 | ንሕቶ ቁ 394 መልስኹም/ኽን እወ እንተኾይኑ ኣብ 2009 ዓ/ም ድሕሪ ግምገማ ሞት ሕንጦታት ክንደይ መጠቓለሊ ሪፖርት ሊእኩም? **( Annex 6 B: ደኩመንት ይርኣዩ)** | | ናብ ክልል……(ቁፅሪ) |  | |  | |
|  |  |  | ናብ ወረዳ……(ቁፅሪ) |  |  |  |  |
|  |  |  | ናብ ጥዕና ትካል……(ቁፅሪ) |  |  |  |  |
| 396 | ንሕቶ 394 መልስኹም/ኽን እወ እንተኾይኑ ገምጋም ምኽንያት ሞት ሕንጦታት ድሕሪ ክንደይ ሰሙን ተላእኹ?( **Annex 6B**) | | ኣብ ዉሽጢ ሓደ ሰሙን……(ቁፅሪ) |  | |  | |
|  |  |  | ድሕሪ ሓደ ሰሙን……(ቁፅሪ) |  |  |  |  |
| 397 | ቁ 394 መልስኹም/ክን እወ እንተኾይኑ ካብቶም ዝተለኣኹ መጠቓለሊ ሞይቶም ንዝተወለዱን ምስ ተወለዱ ንዝሞቱን ሕንጦታት ሪፖርት (**Annex 6B:** Summaryperinatal Death Report) ሙሉእነት ዝጎደሎም ቕጥዕታት ኣለዉ ዶ? **(ምጉዳል ሙሉእነት ቕጥዕታት ረአ)** | | 1= እወ  2= ኣይፋል | መልሲ2→399 | |  | |
| 398 | ንሕቶ 397 መልስኹም/ክን እወ እንተኾይኑ ኣብ 2009 ዓ/ም ክንደይ ፎርማት ጉድለት ምሉእነት ዘለዎም ኣለዉ? | | ……(ቁፅሪ) |  | |  | |
| **ሃላዋት መዛግብትን ቕጥዕታትን ዝምልከት መሕትታት(ብምርኣይ ጥራሕ ዝምለሱ ሕቶታት)** | | | | | | | |
| 399 | ናይ ጭምጭምታ መዝገብ ኣሎ ዶ? | 1= እወ  2= ኣይፋል | | |  | |  |
| 401 | እዞም ዝስዕቡ ልጋባት/ቕጥዕታት ምህላዎም ብምርግጋፅ ዘለዉ ኩሎም ይከበበሎም | 1 =ሞት ኣዶመፍለጢ ቅጥዒ /Maternal Death Notificationformat  2=ሞት ሕንጦመፍለጢ ቅጥዒ/ Perinatal Death Notification format  3=ምክንያት ሞት ኣዶ ድሕሪ ሞት መመርመሪ ቅጥዒ/Maternal verbal Autopsy format  4=ምክንያት ሞት ሕንጦ ድሕሪ ሞት መመርመሪ ቅጥዒ/ prenatal verbal Autopsy format  5= ኣብ ጥዕና ትካል ዝሞታ ኣዴታት ሓበሬታ መአከቢ ቕጥዒ/ Maternal Death Facility Based Abstraction format  6=ኣብ ጥዕና ትካል ዝሞቱ ሕንጦታት ሓበሬታ መአከቢ ቕጥዒ / prenatal Death Facility Based Abstraction format  7=መጠቃለሊ ናይ ሞት ኣዴታት ሪፖርት መግበሪ ቕጥዒ/Maternal Death Case Based Reportformat  8=መጠቃለሊ ናይ ሞት ሕንጦታት ሪፖርት መግበሪ ቕጥዒ/Perinatal Death Case Based Report format  9=መተግበሪ ትልሚ መውፅኢ ቕጥዒ /Action Plan format  10=ሕንጦታት መከታተሊ ቕጥዒ/ Refferal and Neonatal Tracking tool (Annex 8A and 8B) | | |  | |  |
| 402 | ናይ MPNDSR ጋይድላይን ኣሎ ዶ? | 1= እወ  2= ኣይፋል | | |  | |  |
| 403 | ናይ ሪፖርት እዋናውነት መከታተሊ ኣሎ ዶ? | 1= እወ  2= ኣይፋል | | |  | |  |
| 404 | ናይ ሪፖርት ሙሉእነት መከታተሊ ኣሎ ዶ ? | 1= እወ  2= ኣይፋል | | |  | |  |
| 405 | ናይ ሞት ኣዴታት መመዝገቢ ቅጥዒ ኣሎ ዶ ? | 1= እወ  2= ኣይፋል | | |  | |  |
| 406 | ናይ ሞት ሕንጦያት መመዝገቢ ቅጥዒ ኣሎ ዶ? | 1= እወ  2= ኣይፋል | | |  | |  |
| 407 | ናይ ሰሙናዊ ሪፖርት ቕጥዒ ኣሎ ዶ ? | 1= እወ  2= ኣይፋል | | |  | |  |

**ብጣዕሚ የቐንየለይ!!**
