# Supplementary material for: Maternal and perinatal death surveillance and response in Ethiopia: Achievements, challenges and prospects
Source: PLoS One. 2019 Oct 11;14(10):e0223540. doi: 10.1371/journal.pone.0223540 (PMC6788713; doi:10.1371/journal.pone.0223540)
Supplement: S3 File — (DOCX) [file pone.0223540.s004.docx]

**Qualitative Questionnaire for WDG**

**Part I: General Information**

District type…………………

Number of participants………

**Part II: MPDSR implementation**

1. Could you tell us about what you discuss in your WDG meeting?
2. Could you tell us how do you work in your WDG to increase mother and child health service uptake in your WDG? (ANC, Delivery, PNC, FP and EPI service)
3. How do you address all pregnant mothers and home delivery among your WDG members? And how can you know whether these mothers are using health service or not?
4. What do you do if you have any female reproductive age group death and perinatal death in your WDG?
5. How maternal and perinatal death report could be important? And how do you report to HEW about the death occurred in your WDG?

**ዘተኣዊ መጠይቕ ንኣመራርሓ ልምዓት ጉጅለ ደቂ ኣነስትዮ**

**ክፍሊ ሓደ ፡ ሓፈሻዊ ሓበሬታ**

ዓይነት ወረዳ፡ ……………………

በዝሒ ተሳተፍቲ……………………

**ክፍሊ ክልተ ፡ ኣተገባብራ ኣለሻን ግብረ-መልሲ ምሃብን ንሞት ኣዴታን ሕንጦታን**

1. ኣመራርሓ ልምዓት ጉጅለ ኣብ እትእከባሉ ግዜ እንታይ እንታይ ከም እትመያየጣ ዶ ክትነግራና መኸኣልክን?
2. ኣዴታት ኣብ ግልጋሎት ኣዶታትን ህፃናትን (ግልጋሎት ቅድመ ወሊድ ወሊድ ድህረ-ወሊድ ትልሚ ስድራ ከምኡውን ግልጋሎት ክታበት) ተጠቃምነተን ንኽውስኻ ኣብ ልምዓት ጉጅለ ደቂ ኣነስትዮ እትሰርሕዖም ስራሕቲ እንታይ እንታይ እዮም?
3. ኩለን ዝጠነሳ ኣዴታት ብሙልአን ከምዝተነፀራ ከምኡውን ኣብ ገዛ ንዝወለዳ ኣዴታት ንምፍለጥን እትጥቀማሉ መንገዲ (ሜላ) እንታይ ይኸውን?
4. ኣብ ልምዓት ጉጅለኺ ሞት ኣብ ክሊ ምውላድ ዕድመ ዛላ ጓል ኣነስተይቲ ክምኡውን ሞት ሕንጦ እንተኣጋጢሙትክን እንታይ ትገብራከምእገብራ ዶ ብዝርዝር መገለፅክናልና?
5. ምፅብፃብ ሞት ኣዴታትን ሕንጦታትን እንታይ ጠቐሜታ ይህልዎ ይኸውን? ሞት ኣዳታን ሕንጦታትን ኣብ ዘጋጥመክን እወን ብኸመይ ኢኽን ናብ ሰራሕተኛታት ጥሙር ጥዕና ስድራ ቤት እተፍልጣ?
