# Supplementary material for: Maternal and perinatal death surveillance and response in Ethiopia: Achievements, challenges and prospects
Source: PLoS One. 2019 Oct 11;14(10):e0223540. doi: 10.1371/journal.pone.0223540 (PMC6788713; doi:10.1371/journal.pone.0223540)
Supplement: S4 File — (DOCX) [file pone.0223540.s005.docx]

**Qualitative questionnaire for health facilities and district health offices**

**Part I: General Information**

Sex of the participant: __________

Educational level: _____________

Profession: __________________

Work experience: _____________

Position: ____________________

**Part II: MPDSR implementation**

1. How do you monitor the implementation of MPNDSR in this district? (Timely identification, timely notification, routine analysis of death and proper actions to prevent further death )

2. Do have intersectoral collaboration with other sectors on maternal and perinatal health issues? If yes, how?

3. What challenges do have in implementing MPNDSR in your district?

4. What do you try to implement so as tackle these challenges so far?

5. What do you recommend for successful implementation of the program?

**መሕታት ንቃልኣዊ-መሕታት ጥዕና ትካላትን ኣብያተ ፅሕፈትን**

**ክፍሊ ሓደ ፡ ሓፈሻዊ ሓበሬታ**

ፆታ ተሳታፊ/ ተሳታፊት: __________

ደረጃ ትምህርቲ: _____________

ዓይነት ሞያ: __________________

ስራሕ ልምዲ: _____________

ዘለዎ ሓለፍነት: ____________________

**ክፍሊ ክልተ ፡ ኣተገባብራ ኣለሻን ግብረ-መልሲ ምሃብን ንሞት ኣዴታን ሕንጦታን**

1. ኣተገባብራ ኣለሻን ግብረ-መልሲ ምሃብን ንሞት ኣዴታን ሕንጦታን ከመይ ትከታተልዎ? (እዋናዊ ነፀርታ፡ እዋናዊ ምፍላጥ፡ ኣጠቓቕማ እዋናዊ ሓበሬታን ኣብ እዋኑ ስጉምቲ ስጉምቲ ምውሳድን)

2. ኣብ መንጎ ዝተፈላለያ ሴክተራት ኣብ ኣተገባብራ ኣለሻን ግብረ-መልሲ ምሃብን ንሞት ኣዴታን ሕንጦታን ምትሕግጋዝ ኣለኩም ዶ? እወ እንተኾይኑ ብኸመይ?

3. ኣብ ወረዳኹም ወይ ትካልኩም ንኣተገባብራ ኣለሻን ግብረ-መልሲ ምሃብን ንሞት ኣዴታን ሕንጦታን ዕንቕፋት ዝኾነኩም እንታይ እዩ?

4. ንኣተገባብራ ኣለሻን ግብረ-መልሲ ምሃብን ንሞት ኣዴታን ሕንጦታን ዕንቕፋት እዮም ኢልኩም ንዝነፀርኩሞም ማሕንቖታት ንምፍታሕ ክሳብ ሕዚ ዝወሰድኩሞም ስጉምትታት እንታይ እንታይ እዮም?

5. ፕሮግራም ኣተገባብራ ኣለሻን ግብረ-መልሲ ምሃብን ንሞት ኣዴታን ሕንጦታን ዕዉት ንምግባር እንታይ ክግበር ኣለዎ ትብል/ሊ?
