# Supplementary material for: Maternal and perinatal death surveillance and response in Ethiopia: Achievements, challenges and prospects
Source: PLoS One. 2019 Oct 11;14(10):e0223540. doi: 10.1371/journal.pone.0223540 (PMC6788713; doi:10.1371/journal.pone.0223540)
Supplement: S5 File — (DOCX) [file pone.0223540.s006.docx]

**Qualitative Questionnaire for Regional Health Bureau**

**Part I: General information**

Sex: __________________

Profession: ____________

Work experience: _______

Position: ______________

**Part II: MPDSR implementation**

1. How do monitor the implementation of MPNDSR in the region?(The process of early identification, timely notification, quality of death reviewing and short term and long term responses)

2. How do you evaluate the performance of MPNDSR program in the region? (What changes are observed after the initiation of the program?)

3. What challenges do you have in implementing MPNDSR in the region? (Community, health facility, districts and regional wise)

4. What major activities does the region implement to reverse the challenges mentioned earlier?

5. What do you recommend for more successful implementation of the program?

**መሕታት ንቃልኣዊ-መሕታት ንክልል ክኢላ**

**ክፍሊ ሓደ ፡ ሓፈሻዊ ሓበሬታ**

ፆታ ተሳታፊ/ ተሳታፊት: __________

ደረጃ ትምህርቲ: _____________

ዓይነት ሞያ: __________________

ስራሕ ልምዲ: _____________

ዘለዎ ሓለፍነት: ____________________

**ክልተ ፡ ኣተገባብራ ኣለሻን ግብረ-መልሲ ምሃብን ንሞት ኣዴታን ሕንጦታን**

1. ኣተገባብራ ኣለሻን ግብረ-መልሲ ምሃብን ንሞት ኣዴታን ሕንጦታን ከመይ ትከታተልዎ? (ኣ ከይዲ እዋናዊ ነፀርታ፡ እዋናዊ ምፍላጥ፡ ኣጠቓቕማ እዋናዊ ሓበሬታን ኣብ እዋኑ ስጉምቲ ስጉምቲ ምውሳድን)

2. ኣፈፃፅማ ትገበራ ፕሮግራም ኣለሻን ግብረ-መልሲ ምሃብን ንሞት ኣዴታን ሕንጦታን ከመይ ትግምግሞ/ትግምግምዮ?

3. ኣብዚ ክልል ንኣተገባብራ ኣለሻን ግብረ-መልሲ ምሃብን ንሞት ኣዴታን ሕንጦታን ዕንቕፋት ዝኾነኩም እንታይ እዩ?( ኣብ ማሕበረ ሰብ፡ ኣብ ጥዕና ትካላት፡ ኣብ ወረዳታትን ክልልን)

4. ንኣተገባብራ ኣለሻን ግብረ-መልሲ ምሃብን ንሞት ኣዴታን ሕንጦታን ዕንቕፋት እዮም ኢልኩም ንዝነፀርኩሞም ማሕንቖታት ንምፍታሕ ከም ክልል ክሳብ ሕዚ ዝወሰድኩሞም ስጉምትታት እንታይ እንታይ እዮም?

5. ከም ክልል ክኢላ ፕሮግራም ኣተገባብራ ኣለሻን ግብረ-መልሲ ምሃብን ንሞት ኣዴታን ሕንጦታን ዕዉት ንምግባር እንታይ ክግበር ኣለዎ ትብል/ሊ?
