# Supplementary material for: Maternal and perinatal death surveillance and response in Ethiopia: Achievements, challenges and prospects
Source: PLoS One. 2019 Oct 11;14(10):e0223540. doi: 10.1371/journal.pone.0223540 (PMC6788713; doi:10.1371/journal.pone.0223540)
Supplement: S1 Annex — (DOCX) [file pone.0223540.s007.docx]

| **Variable** | **Value** |
| --- | --- |
| Early identification of maternal death | 1=Yes, 0=No |
| Early identification of perinatal death | 1=Yes, 0=No |
| Reports perinatal death in 2009 E.C | 1=Yes, 0=No |
| Proper means to identify maternal death | 1=Yes, 0=No |
| Proper means to identify perinatal death | 1=Yes, 0=No |
| Availability of weekly zero report on maternal death | 1=Yes, 0=No |
| >=85% proportion of maternal death weekly zero reports | 1=Yes, 0=No |
| Availability of weekly zero report on perinatal death | 1=Yes, 0=No |
| >=85% proportion of perinatal death weekly zero reports | 1=Yes, 0=No |
| WDG sends Weekly maternal and perinatal death reports | 1=Yes, 0=No |
| Availability of weekly zero report for perinatal death from WDA | 1=Yes, 0=No |
| Good quality of death notification for maternal death | 1=Yes, 0=No |
| Good quality of death notification for perinatal death | 1=Yes, 0=No |

**Annex 1: Variables computed for the quality of early maternal and/or perinatal death identification.**
