# Supplementary material for: Maternal and perinatal death surveillance and response in Ethiopia: Achievements, challenges and prospects
Source: PLoS One. 2019 Oct 11;14(10):e0223540. doi: 10.1371/journal.pone.0223540 (PMC6788713; doi:10.1371/journal.pone.0223540)
Supplement: S2 Annex — (DOCX) [file pone.0223540.s008.docx]

**Annex 2: Variables computed for the quality of death review.**

| **Variable** | **Value** |
| --- | --- |
| Did the health facility take FBDA for maternal death? | 1=Yes, 0=No |
| No missed variable in the Maternal FBDA formant | 1=Yes, 0=No |
| Did the health facility receive maternal VA? | 1=Yes, 0=No |
| Maternal VA received within 3-4 week | 1=Yes, 0=No |
| No Maternal VA received after 4 weeks | 1=Yes, 0=No |
| No maternal VA with missed variable | 1=Yes, 0=No |
| Did the HF reviews for maternal death | 1=Yes, 0=No |
| Did the health facility take perinatal FBDA | 1=Yes, 0=No |
| No missed variable in perinatal FBDA | 1=Yes, 0=No |
| Did the health facility receive perinatal VA | 1=Yes, 0=No |
| Did the health facility review for perinatal death | 1=Yes, 0=No |
| Is there proper document handling in the HF? | 1=Yes, 0=No |
| Did the health facility notify maternal death to District Health office? | 1=Yes, 0=No |
| There is no missed variable in the maternal notification formant | 1=Yes, 0=No |
| Did the facility PHEM notify perinatal death formally? | 1=Yes, 0=No |
| There is no missed variable in the perinatal notification formant | 1=Yes, 0=No |
| Did HF send maternal death summery report? | 1=Yes, 0=No |
| There is no maternal death summery report with missed variable | 1=Yes, 0=No |
| Did the HF sent perinatal death summery report? | 1=Yes, 0=No |
| There is no missed variable in the perinatal death summery report | 1=Yes, 0=No |
