# Supplementary material for: Maternal and perinatal death surveillance and response in Ethiopia: Achievements, challenges and prospects
Source: PLoS One. 2019 Oct 11;14(10):e0223540. doi: 10.1371/journal.pone.0223540 (PMC6788713; doi:10.1371/journal.pone.0223540)
Supplement: S3 Annex — (DOCX) [file pone.0223540.s009.docx]

**Annex 3: Variables computed for the proper response implementation.**

| **Variable** | **Value** |
| --- | --- |
| Was action plan developed for reviewed maternal death? | 1=Yes, 0=No |
| Was action plan developed for perinatal death? | 1=Yes, 0=No |
| Was the facility made discussion with staff? | 1=Yes, 0=No |
| Was the health facility conducted on job training? | 1=Yes, 0=No |
| Was the health facility improved medical supply availability? | 1=Yes, 0=No |
| Was the health facility construct maternity waiting room? | 1=Yes, 0=No |
| Was the health facility made arrangement of layout of health facility? | 1=Yes, 0=No |
| Was the health facility took administrative corrective measures? | 1=Yes, 0=No |
| Did the health facility discussed with WDA about MPNDSR? | 1=Yes, 0=No |
| Did the health facility discussed with WDA about ANC? | 1=Yes, 0=No |
| Did the health facility discussed with WDA about skilled delivery? | 1=Yes, 0=No |
| Did the health facility discussed with WDA about PNC? | 1=Yes, 0=No |
| Discussed on importance of birth preparedness and complication readiness? | 1=Yes, 0=No |
| Did the health facility discussed with WDA about danger sign during pregnancy? | 1=Yes, 0=No |
| Did the health facility discussed with WDA about perinatal period danger sign? | 1=Yes, 0=No |
| Are the MPNDSR activities incorporated as part of annual plan? | 1=Yes, 0=No |
