# Supplementary material for: Maternal and perinatal death surveillance and response in Ethiopia: Achievements, challenges and prospects
Source: PLoS One. 2019 Oct 11;14(10):e0223540. doi: 10.1371/journal.pone.0223540 (PMC6788713; doi:10.1371/journal.pone.0223540)
Supplement: S1 Appendex — (DOC) [file pone.0223540.s010.doc]

**STROBE Statement—Checklist of items that should be included in reports of *cross-sectional studies* on Maternal and Perinatal Death Surveillance and Response in Ethiopia: Achievements, challenges and prospects.**

|  | Item No | Recommendation |
| --- | --- | --- |
| **Title and abstract** | 1 | (*a*) Indicate the study’s design with a commonly used term in the title or the abstract  Abstract: Line 25 and 26 |
|  |  | (*b*) Provide in the abstract an informative and balanced summary of what was done and what was found. Abstract: paragraphs 2, 3 and 4 |
| Introduction | | |
| Background/rationale | 2 | Explain the scientific background and rationale for the investigation being reported  Introduction: paragraph 9-10 |
| Objectives | 3 | State specific objectives, including any pre specified hypotheses  Introduction: Line 131-132 |
| Methods | | |
| Study design | 4 | Present key elements of study design early in the paper  Methods: paragraph 2 |
| Setting | 5 | Describe the setting, locations, and relevant dates, including periods of recruitment, exposure, follow-up, and data collection. Methods: paragraph 1and 2 |
| Participants | 6 | (*a*) Give the eligibility criteria, and the sources and methods of selection of participants Methods: paragraph 3-4 |
| Variables | 7 | Clearly define all outcomes, exposures, predictors, potential confounders, and effect modifiers. Give diagnostic criteria, if applicable. Methods: Measurement of variables line 219-267 |
| Data sources/ measurement | 8* | For each variable of interest, give sources of data and details of methods of assessment (measurement). Describe comparability of assessment methods if there is more than one group Methods: Data collection tool and technique line 171-217 |
| Bias | 9 | Describe any efforts to address potential sources of bias Methods section: Data quality control measures line 273-280 |
| Study size | 10 | Explain how the study size was arrived at. Methods: Sampling procedure and participant requirement line 150-156 |
| Quantitative variables | 11 | Explain how quantitative variables were handled in the analyses. If applicable, describe which groupings were chosen and why Methods: Data management and analysis line 282-289 |
| Statistical methods | 12 | (*a*) Describe all statistical methods, including those used to control for confounding Methods: Data management and analysis line 290-294 |
|  |  | (*b*) Describe any methods used to examine subgroups and interactions |
|  |  | (*c*) Explain how missing data were addressed. |
|  |  | (*d*) If applicable, describe analytical methods taking account of sampling strategy |
|  |  | (*e*) Describe any sensitivity analyses |
| Results | | |
| Participants | 13* | (a) Report numbers of individuals at each stage of study—eg numbers potentially eligible, examined for eligibility, confirmed eligible, included in the study, completing follow-up, and analysed. Result: paragraph 1 |
|  |  | (b) Give reasons for non-participation at each stage |
|  |  | (c) Consider use of a flow diagram |
| Descriptive data | 14* | (a) Give characteristics of study participants (eg demographic, clinical, social) and information on exposures and potential confounders Result: quantitative finding paragraph 1and qualitative finding paragraph 1 |
|  |  | (b) Indicate number of participants with missing data for each variable of interest Result: Table 2-5 |
| Outcome data | 15* | Report numbers of outcome events or summary measures Result: paragraph 2,4 and 5 |
| Main results | 16 | (*a*) Give unadjusted estimates and, if applicable, confounder-adjusted estimates and their precision (eg, 95% confidence interval). Make clear which confounders were adjusted for and why they were included Result: Factors associated with MPDSR implementation: paragraph 1 and 2, tables 6-8 |
|  |  | (*b*) Report category boundaries when continuous variables were categorized |
|  |  | (*c*) If relevant, consider translating estimates of relative risk into absolute risk for a meaningful time period |
| Other analyses | 17 | Report other analyses done—eg analyses of subgroups and interactions, and sensitivity analyses |
| Discussion | | |
| Key results | 18 | Summarise key results with reference to study objectives  Discussion : paragraph 1 to13 |
| Limitations | 19 | Discuss limitations of the study, taking into account sources of potential bias or imprecision. Discuss both direction and magnitude of any potential bias  Discussion :Strength and limitation: paragraph 1 |
| Interpretation | 20 | Give a cautious overall interpretation of results considering objectives, limitations, multiplicity of analyses, results from similar studies, and other relevant evidence  Conclusion : paragraph 1and 2 |
| Generalizability | 21 | Discuss the generalizability (external validity) of the study results |
| Other information | | |
| Funding | 22 | Give the source of funding and the role of the funders for the present study and, if applicable, for the original study on which the present article is based  In online submission form: The study was funded by United Nations Population Fund Agency (UNFPA) in collaboration with Tigray Regional Health Bureau. The funder had no role in study design, data collection and analysis, and preparation of the manuscript. |

*Give information separately for exposed and unexposed groups.

**Note:** An Explanation and Elaboration article discusses each checklist item and gives methodological background and published examples of transparent reporting. The STROBE checklist is best used in conjunction with this article (freely available on the Web sites of PLoS Medicine at http://www.plosmedicine.org/, Annals of Internal Medicine at http://www.annals.org/, and Epidemiology at http://www.epidem.com/). Information on the STROBE Initiative is available at www.strobe-statement.org.
